# Molecular insights into the role of desmin intermediate filament network in chromatin landscape, cardiomyocyte differentiation, and maturation

**DOI:** 10.1038/s41419-025-08056-3

**Published:** 2025-10-16

**Authors:** Mary Tsikitis, Antigoni Diokmetzidou, Panagiotis Liakopoulos, Maria Karipidou, Ioannis Kokkinopoulos, Giannis Vatsellas, Ismini Kloukina, Petros Kolovos, Yassemi Capetanaki

**Affiliations:** 1 Center of Basic Research, Biomedical Research Foundation, Academy of Athens, Athens, Greece; 2 Department of Biology, University of Padua, Padova, Italy; 3 Veneto Institute of Molecular Medicine, Padova, Italy; 4 Department of Molecular Biology and Genetics, Democritus University of Thrace, Alexandroupolis, Greece; 5 Genome Center, Biomedical Research Foundation, Academy of Athens, Athens, Greece

**Keywords:** Intermediate filaments, Nuclear organization

## Abstract

The cardiac cytoskeleton is essential for the proper intracellular and intercellular integration of cardiomyocyte structure, maintenance and function, failure of which leads to cell death and heart disease. In particular, the desmin intermediate filament cytoskeleton, due to its early expression and subcellular distribution, enables the formation of a continuous network that connects the extracellular space with the nuclear matrix, thus allowing us to hypothesize potential key roles in mechanotransduction to the nucleus, and consequently, in cardiac lineage determination, differentiation and development. Here, we investigated the role of desmin in cardiac differentiation and homeostasis across embryogenesis, postnatal life, and adulthood, as well as under various stimuli. Desmin was found to be highly expressed in cardiac progenitor cells during embryogenesis, and its absence significantly impacts the size of this population. Using direct cellular reprogramming of fibroblasts to induced cardiomyocytes, we demonstrated that ectopic desmin expression directly influences cardiomyogenesis, potentially linked, among others, to transcriptional regulation of the Notch1 signaling. Conversely, desmin deficiency leads to substantial perturbations in cardiac maturation by diminishing the expression and proper localization of cardiac-specific proteins, impairing calcium homeostasis and delaying myofibril formation. RNA and ChIP sequencing, coupled with Hi-C analysis, revealed that desmin depletion causes chromatin architectural disruptions, altering genome organization and gene expression of cardiac-specific modulators, contributing to pathophysiological phenotype.

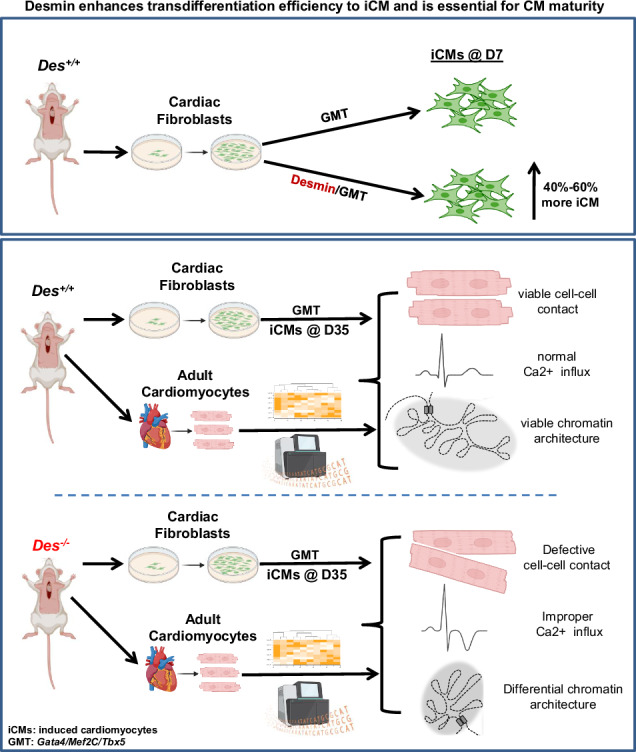

## Introduction

Intermediate filaments (IFs) are a fundamental component of the vertebrate cytoskeleton. Initially regarded as solely structural and mechanical elements [1], genetic and experimental evidence have revealed broader and diverse regulatory roles, notably in skeletal and cardiac development and differentiation [2, 3]. In mature striated muscle, the IF network is primarily composed of the muscle-specific protein desmin, with smaller contributions from non-muscle IFs and nuclear lamins. Desmin, along with its multiple binding partners [4], forms a three-dimensional scaffold connecting the contractile apparatus to costameres, intercalated discs, and intracellular organelles, including mitochondria, the sarcoplasmic reticulum and the nucleus [2, 5]. Of particular interest for the present work is the association of desmin with nuclear lamins either directly through the nuclear pores [6, 7] or indirectly through plectin-nesprin3-LINC (linker of the nucleoskeleton and the cytoskeleton) complex [2, 5, 8, 9]. This linkage connects the extracellular matrix to the nuclear lamina, suggesting a functional interplay with chromatin architecture, genome organization and gene expression [2, 5, 9–12]. Mutations in desmin and lamin genes result in myopathies, called desminopathies and laminopathies [2, 5] respectively, which are associated with conduction system malfunction and multiple forms of cardiomyopathy (CM), most frequently with dilated cardiomyopathy (DCM), leading to progressive myocardial degeneration and heart failure [2, 13–15]. We and others have previously demonstrated that desmin maintains mitochondrial integrity, preventing cardiomyocyte degeneration, inflammation and fibrosis [2, 13–19]. In vitro findings highlight desmin’s involvement in myogenesis and cardiogenesis [2, 20–24], prompting questions regarding its in vivo role in cardiac development. During mouse embryogenesis, desmin is among the earliest muscle-specific proteins detected, appearing at 8.25 days postcoitum (d.p.c.) in the primitive cardiac tube, at 8.5 d.p.c. in the heart rudiment and at 9 d.p.c. in the myotome of the somites [20, 25, 26], preceding all muscle-specific structural genes and myogenic transcription regulators [20, 21, 27, 28]. Here, we identify desmin expression in cardiac progenitor cells (CPCs) as early as E7.5, preceding previously reported timelines and suggesting roles in early lineage specification and cytoskeletal network organization. Notably, desmin is also expressed at low levels in adult skeletal muscle progenitors (satellite cells) [29] and subsets of cardiac stem cells (side population, or SP cells, and Sca1+ cells) [30], with transient nuclear localization observed in embryonic stem cell-derived CPCs [31]. The uniqueness of desmin expression in both embryogenesis and adulthood suggests key roles in mechanotransduction and development, transmitting extracellular mechanical forces to nuclear gene-regulatory machinery through interactions with lamins. Such mechanosensitivity is relevant to cardiomyogenesis, which integrates transcriptional programs with hemodynamic forces such as blood flow [32, 33]. However, the precise role of early desmin expression during development remains uncertain, particularly regarding its potential influence on cardiac transcriptional programs and preferential effects on specific CPC subpopulations during development.

In this study, we aimed to identify the role of early desmin expression during development, particularly its impact on cardiac transcriptional programs. We applied direct reprogramming (transdifferentiation) of cardiac fibroblasts into induced cardiomyocytes (iCMs) by retroviral delivery of Gata4, Mef2c, and Tbx5 (GMT) [34–36]. Presence of desmin increased reprogramming efficiency by ~50% (±10%), partly through early activation of the Notch1 pathway, a central regulator of cardiac development, differentiation, and regeneration [37, 38]. Integration of in vitro and in vivo analyses revealed that loss of desmin impaired CPC viability, disrupted in vivo cardiac development, and hindered iCM maturation by modulating expression and localization of cardiac-specific proteins during maturation. Genome-wide chromatin profiling further demonstrated that desmin deficiency alters local chromatin architecture, predominantly during developmental transitions and under stress. These findings provide a novel mechanistic framework for how a cytoskeletal protein can directly influence nuclear organization and transcriptional programs, offering new insights into the pathogenesis of desminopathies and advancing the cardiac regeneration field.

Together, our results dissect and illuminate the comprehensive role played by desmin IF network in chromatin landscape, cardiogenic cell determination, differentiation and maturation during cardiac development.

## Results

### Desmin enhances cardiac trans-differentiation by Gata4, Mef2c, and Tbx5 reprogramming factors

**Fig. 1:**
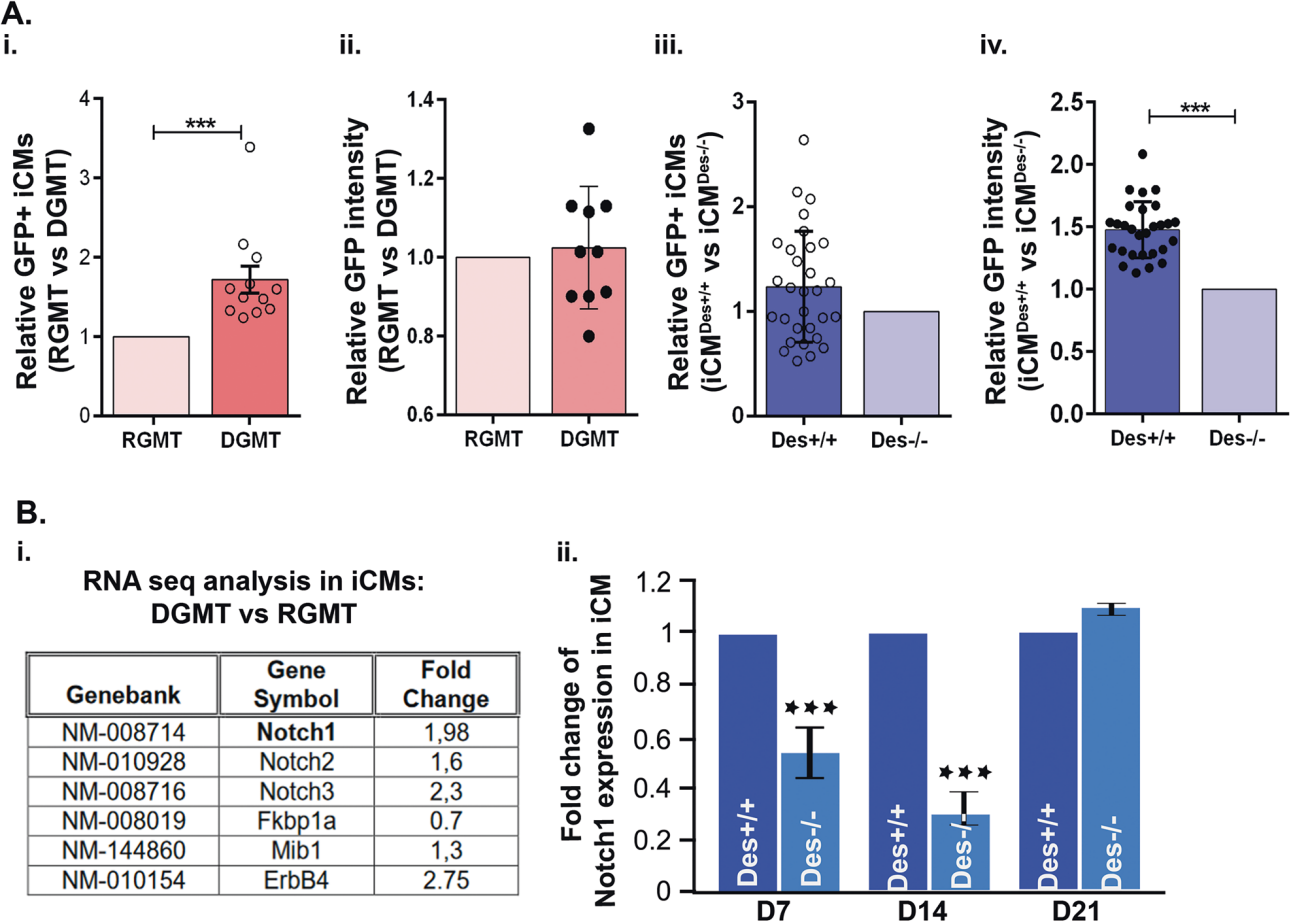
Addition of desmin in the GMT cocktail enhances cardiac trans-differentiation and increases the expression of Notch pathway proteins. **A** FACS analysis for α-MHC-GFP^+^ cells on day 7 (D7) after transduction. i) Fold increase of the GFP^+^ cells (iCMs) after desmin addition to GMT (DGMT) cocktail vs. addition of DsRED (control) (RGMT), *n* = 20. ii) Comparison of the mean intensity of GFP signal in iCMs from (i). iii) Fold difference of the percentage of GFP^+^ cells from transdifferentiated fibroblasts derived from *Des*^*+/+*^vs. *Des*^*−/−*^ mouse hearts (iCMs^*Des+/+*^ vs. iCMs^*Des−/−*^) (*n* = 20). iv) Comparison of the mean intensity of GFP signal in cells from (iii). **B** RNA sequencing and relative mRNA expression analysis. i) Table of RNA sequencing results of iCMs obtained from DGMT vs. control RGMT transduction showing differential expression of Notch pathway genes. ii) Relative Notch1 mRNA expression levels by qPCR analysis of iCMs^*Des+/+*^ vs. iCMs^*Des−/−*^ at different time points following transduction with GMT cocktail. DGMT: Desmin-Gata4-Mef2c-Tbx5, RGMT: DsRed-Gata4-Mef2c-Tbx5. iCMs: induced cardiomyocytes. Statistics by Student’s *t*-test. All data are presented as means ± SEM. **p < 0.05; ***p < 0.005 vs. relevant control.

To better understand the role of desmin during early cardiogenesis, we set out to determine if it influences the initial steps of cardiac determination. A direct reprogramming approach, transdifferentiation, was employed, whereby cardiac fibroblasts (CFs) isolated from 1–3-day-old MHC-GFP transgenic mice were converted into induced cardiomyocytes (iCMs) via retroviral delivery of the transcription factors Gata4, Mef2c, and Tbx5 (GMT) [34–36]. The MHC-GFP transgenic mouse line used expresses GFP under a cardiac-specific myosin heavy chain (MHC) (Myh6) promoter to distinguish GFP(+) cardiomyocytes [34] from the GFP(-) fibroblasts. CFs inherently lack GFP and desmin expression, as both are under cardiac-specific promoters. CFs from desmin-expressing mice, *MYH*^*GFP/GFP*^*/Des*^*+/+*^ (*Des*^*+/+*^), were successfully reprogrammed into iCMs expressing GFP as early as day 3 after transduction, as previously reported [34]. Flow cytometry analysis revealed that the addition of desmin to the GMT (DGMT) cocktail increased the trans-differentiation efficiency of CFs into iCMs by 50% ± 10% by day seven (D7) after transduction when compared to a control DsRed-GMT (RGMT) cocktail (Fig. 1Ai). These results demonstrate that desmin impacts early cardiomyocyte commitment. While there was an enhancement of trans-differentiation, no significant difference in GFP fluorescence intensity was observed (Fig. 1Aii), indicating that endogenous desmin expression in both RGMT- and DGMT-treated iCMs by days 3–4 was sufficient to achieve maximal Myh6 promoter-driven GFP output.

To assess the consequences of desmin deficiency in early trans-differentiation, we generated *MYH*^*GFP/GFP*^ desmin-deficient mice (*Des*^*−/−*^). CFs from 1–3-day-old *Des*^*+/+*^ and *Des*^*−/−*^ mice were reprogrammed with the GMT viral cocktail. Flow cytometry analysis on D7 revealed no difference between genotypes in the percentage of GFP-expressing iCMs (Fig. 1Aiii), as expected, given the absence of desmin expression in both CF types, resulting in a comparable initiation of trans-differentiation across both genetic backgrounds. This is consistent with prior reports indicating that the decisive mechanisms governing cardiac reprogramming occur within the first 72 h after induction [39, 40]. However, the mean intensity of the GFP signals which corresponds to MHC expression levels, was 50% higher in iCMs^*Des+/+*^ derived from fibroblasts isolated from *Des*^*+/+*^ mice compared to the ones (iCMs^*Des−/−*^) originated from the *Des*^*−/−*^ mice (Fig. 1Aiv), implying that desmin, in addition to its very early impact in cardiomyocyte commitment, influences cardiac specific MHC expression during early maturation stages.

### Desmin positively regulates the Notch pathway during early cardiogenesis

To elucidate the potential mechanisms by which desmin enhances the cardiomyocyte trans-differentiation process, transcriptomic analysis (RNA-seq) was performed on iCMs at D7 following DGMT transduction and compared with RGMT control. This revealed that ectopic expression of desmin altered, among others, the expression of the *Notch* pathway members (Fig. 1Bi). Specifically, *Notch1* and *Notch3* expression increased two-fold, and *Notch2* 1.6-fold, while *Fkbp1a*, a cis-trans prolyl isomerase recently identified as a novel negative modulator of activated Notch1 [41], was downregulated in the DGMT iCMs, suggesting that the Notch pathway is potentially both directly and indirectly regulated by desmin. To assess the requirement for endogenous desmin, *Notch* gene expression profiles were compared between iCMs^*Des−/−*^ and iCMs^*Des+/+*^. A marked reduction was observed in iCMs^*Des−/−*^, persisting for two weeks (D14) but resolving by D21 (Fig. 1Bii), corroborating the need for desmin for proper *Notch* expression early in development, mainly through temporal regulation.

Given the established role of the Notch pathway in cell differentiation, tissue development, and regeneration [42], its regulation by desmin was further examined in adult mouse hearts under basal and stress conditions. Using a swimming exercise model of mild muscular stress, cardiac Notch1 mRNA and protein levels were quantified in *Des*^*+/+*^ and *Des*^*−/−*^ adult mice. While basal levels were comparable between genotypes, Notch1 mRNA and protein levels were markedly reduced in *Des*^*−/−*^ adult hearts following stress exposure (Fig. S1). Driven by scientific curiosity, we evaluated the Hippo pathway effector YAP, also implicated in cardiac regeneration [43]. Although YAP mRNA levels were unaffected, protein levels were reduced in desmin-deficient hearts following stress, suggesting impairment of multiple regeneration-associated pathways in the absence of desmin (Fig. S1).

Meanwhile, RNA-seq analysis further revealed differential expression of *Mib1* [44] and *ErbB4* [45], which are both involved in trabecular maturation and compaction by modulating the Notch1 signaling pathway, upon ectopic desmin expression (Fig. 1Bi).

### Lack of desmin causes cardiac developmental defects

**Fig. 2:**
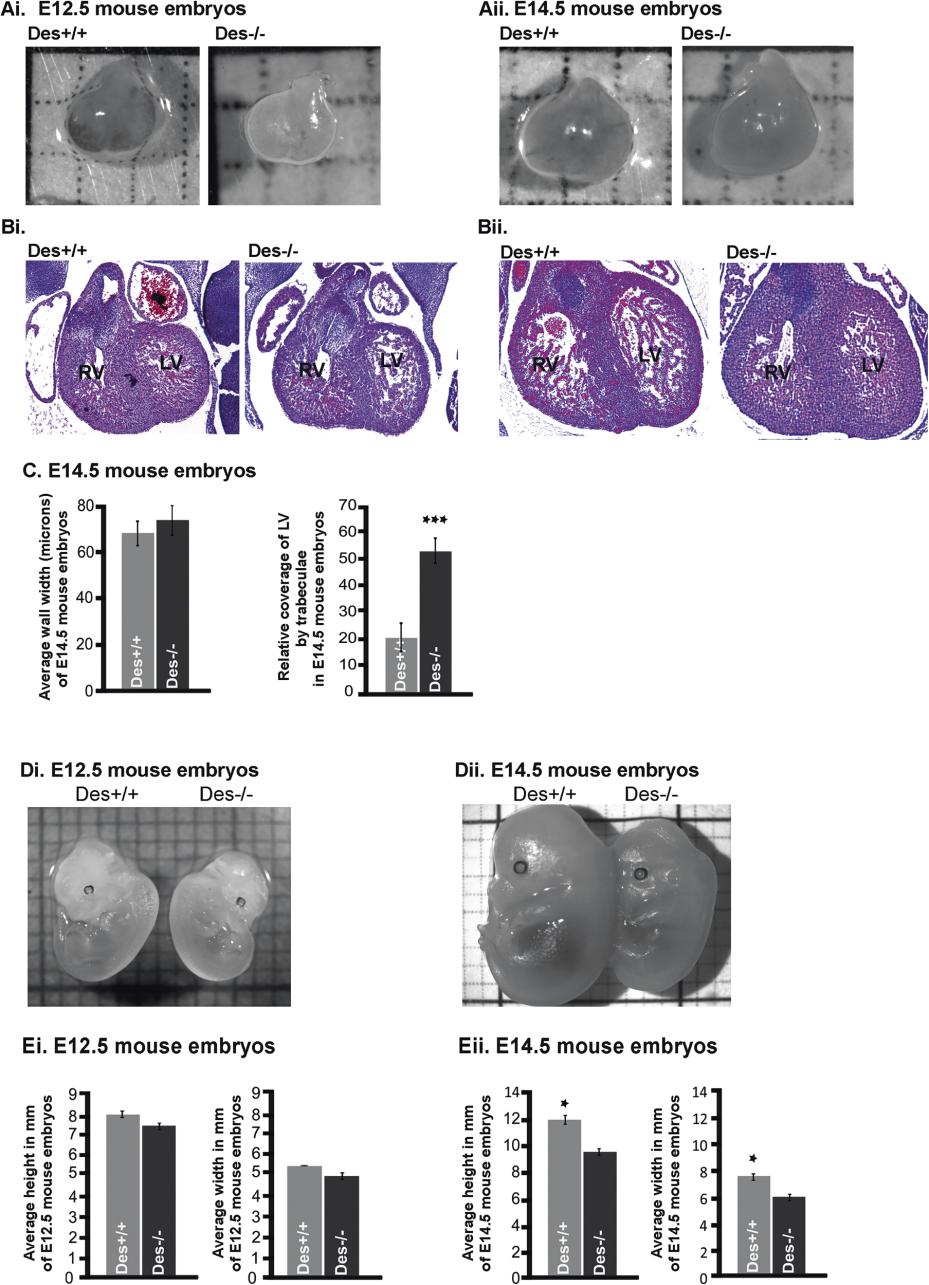
Desmin deficiency leads to defects in cardiac development and embryonic body size. **A** Stereoscopic analysis of *Des*^*+/+*^ and *Des*^*−/−*^ hearts at embryonic day E12.5 (Ai: *n* = 7 *Des*^*+/+*^ and *n* = 8 *Des*^*−/−*^) and E14.5 (Aii: *n* = 7 Des^*+/+*^ and Des^*−/−*^). **B** Histological analysis of hematoxylin & eosin-stained representative paraffin sections of E12.5 (Bi: *n* = 3 *Des*^*+/+*^ and *Des*^*−/−*^) and E14.5 hearts (Bii: *n* = 4 *Des*^*+/+*^and *Des*^*−/−*^) of *Des*^*+/+*^and *Des*^*−/−*^ embryos. RV: right ventricle, LV: left ventricle. **C** Quantitation of cardiac trabeculae and cardiac wall thickness of the left ventricle of E14.5 embryonic hearts. (*n* = 11 *Des*^*+/+*^and Des^*−/−*^). **D** Stereoscopic analysis of Des^+/+^ and Des^*−/−*^ whole body at embryonic day Di) E12.5 (*n* = 7 *Des*^*+/+*^ and *n* = 6 *Des*^*−/−*^) and Dii) E14.5 (*n* = 7 *Des*^*+/+*^and *Des*^*−/−*^). **E** Quantitation of body width and height (measured in millimeters) at embryonic day E12.5 (Ei) and E14.5 (Eii) (right panels). Statistical analysis by unpaired Student’s *t*-test. All data are presented as means ± SEM. *p < 0.005 of *Des*^*+/+*^ vs*. Des*^*−/−*^.

Desmin’s temporal control on the Notch pathway, alongside modulation of *Mib1* and *ErbB4*, could impact cardiac specification, progenitor cell differentiation, and ventricular trabeculation and compaction [38]. To test the latter, hearts from *Des*^*+/+*^ and *Des*^*−/−*^ embryos were examined in situ. Stereological analyses at E12.5 and E14.5 showed no major gross morphological differences (Fig. 2Ai, Aii). Histological analysis at E12.5 showed no statistical differences between the two backgrounds (Fig. 2Bi); however, *Des*^*−/−*^ hearts at E14.5 revealed a significant increase in trabeculae number, with structures appearing disorganized, non-compacted, and extending into the ventricular lumen, indicative of defective trabecular maturation (Fig. 2Bii, C). Additionally, morphometric measurements demonstrated reduced *Des*^−/−^ embryo size at E12.5, 6.25% shorter and 20% narrower than controls, and at E14.5, 20% shorter and 25% narrower (Fig. 2D, E).

### Desmin is expressed in Cardiac Progenitor Cells (CPCs) and regulates the size of their population

**Fig. 3:**
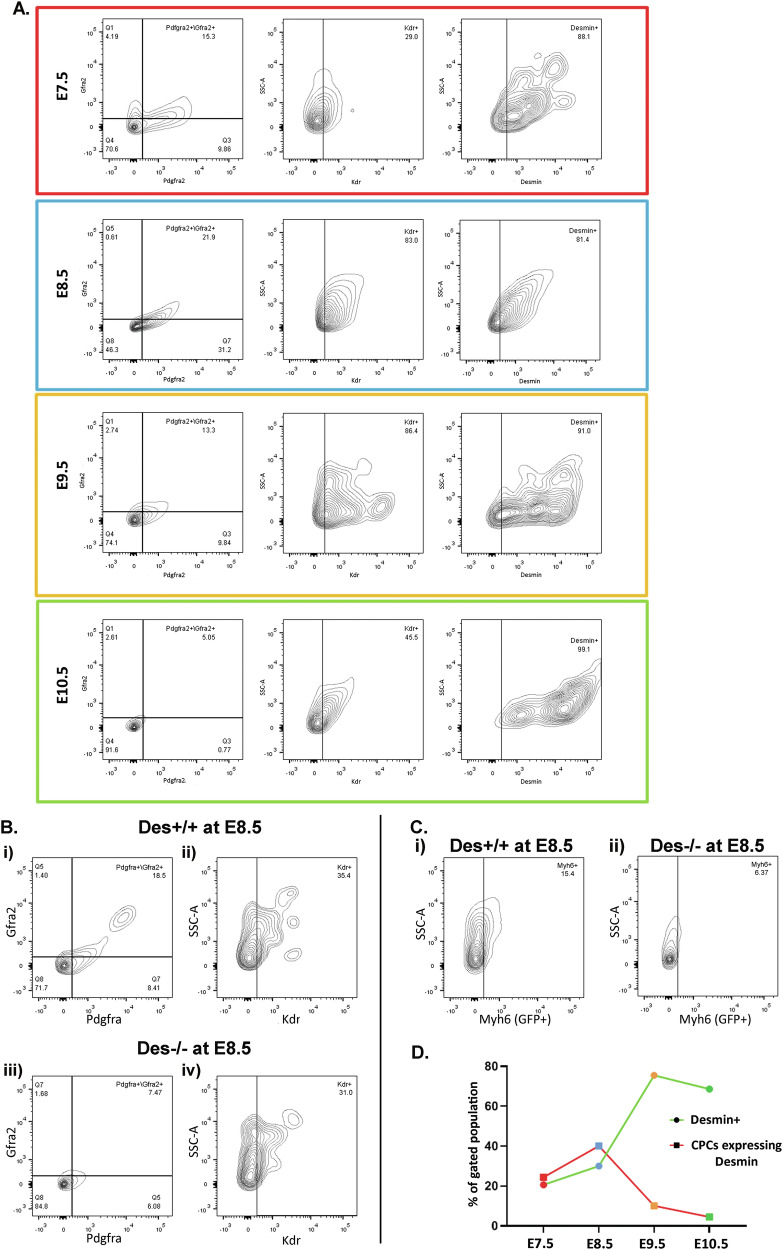
Desmin is expressed in the *Pdgfra*^*+*^*/Gfra2*^*+*^*/Kdr*^*+*^ CPC population and regulates its size. **A** FACS analyses of desmin expression in *Pdgfra*^*+*^*/Gfra2*^*+*^*/Kdr*^*+*^ CPCs isolated from mouse hearts at embryonic days E7.5 (*n* = 2), E8.5 (*n* = 3), E9.5 (*n* = 3) and E10.5 (*n* = 2). The first plot presents the cardiac cell population according to *Pdgfra*^*+*^*/Gfra2*^*+*^ expression. The second plot shows the percentage of *Kdr*^*+*^ cells in the *Pdgfra*^*+*^*/Gfra2*^*+*^ population, and the third plot shows the percentage of *Pdgfra*^*+*^*/Gfra2*^*+*^*/Kdr*^*+*^ population that expresses desmin. **B** FACS plots of CPCs isolated from *Des*^*+/+*^ (Bi and Bii) vs. *Des*^*−/−*^ (Biii and Biv) mouse hearts at E8.5, *n* = 2. The first plot presents the cardiac cell population according to *Pdgfra*^*+*^*/Gfra2*^*+*^ expression. The second plot shows the percentage of *Kdr*^*+*^ cells in the *Pdgfra*^*+*^*/Gfra2*^*+*^ population, that is, the triple-positive *Pdgfra*^*+*^*/Gfra2*^*+*^*/Kdr*^*+*^ CPCs. **C** FACS plots showing the percentage of MHC (Myh6) in the total cardiac cell population of *Des*^*+/+*^ (**Ci**) and *Des*^*−/−*^ (**Cii**) of E8.5 mouse embryonic hearts. **D** Percentage of desmin+ cells in total cardiac cell population; the line with circles represents the percentage of cardiac cells that express desmin during E7.5-E10.5, the line with squares represents the percentage of CPCs that express desmin. CPCs cardiac progenitor cells.

Cardiac progenitor cells (CPCs) are essential for normal heart development. To determine whether desmin is expressed in CPCs and whether its absence contributes to the cardiac defects observed in *Des*^*−/−*^ mice, we isolated cardiac cells from the cardiac crescent/first heart field (FHF) and second heart field (SHF) at E7.5, and from whole embryo hearts at E8.5, E9.5, and E10.5. CPCs were identified by FACS using triple labeling for *Pdgfra*, *Kdr* (*Vegfr*), and *Gfra2*, which specifically mark FHF and SHF CPCs [46, 47]. The triple-positive CPC fraction represented 12.7 ± 5.4% of total cardiac cells at E8.5, declining to 10.7 ± 4.1% at E9.5 and 2.1 ± 0.5% at E10.5, consistent with progressive differentiation and concomitant dwindling of the stem cell population (Fig. 3A). Desmin immunolabeling in the flow cytometry analysis confirmed desmin expression in >90% of CPCs at all stages, indicating it as a reliable marker of *PDGFRa* +*/GFRa2* +*/Kdr* + CPCs (Fig. 3A, S2A). A developmental shift from low to high desmin expression was observed in total cardiac cells from E7.5 to E10.5 (Fig. S2A). At the same time, the proportion of desmin-positive CPCs decreased, reflecting their maturation into cardiomyocytes (Fig. 3D and Fig. S2B).

We next tested CPC population fate in the absence of desmin. CPCs from *Des*^*+/+*^ and *Des*^*−/−*^ E8.5 hearts were compared. Desmin loss reduced the triple-positive CPC fraction by >50% (6.55% in Des + /+ vs. 2.32% in Des−/−, *p* < 0.1) (Fig. 3B panels i-ii vs. iii-iv), indicating impaired CPC viability and/or self-renewal. Additionally, GFP reporter analysis revealed a nearly threefold reduction in GFP-positive cells within the total cardiac population of *Des*^*−/−*^ embryos (6.41% vs. 15.5% in Des + /+, *p* < 0.005) (Fig. 3C). This reduction persisted at later stages, likely reflecting both delayed CPC commitment to a cardiomyocyte-like fate and retarded maturation. These findings position desmin as a critical determinant of CPC maintenance and timely differentiation during early cardiogenesis.

### Desmin is important for cardiomyocyte maturation

**Fig. 4:**
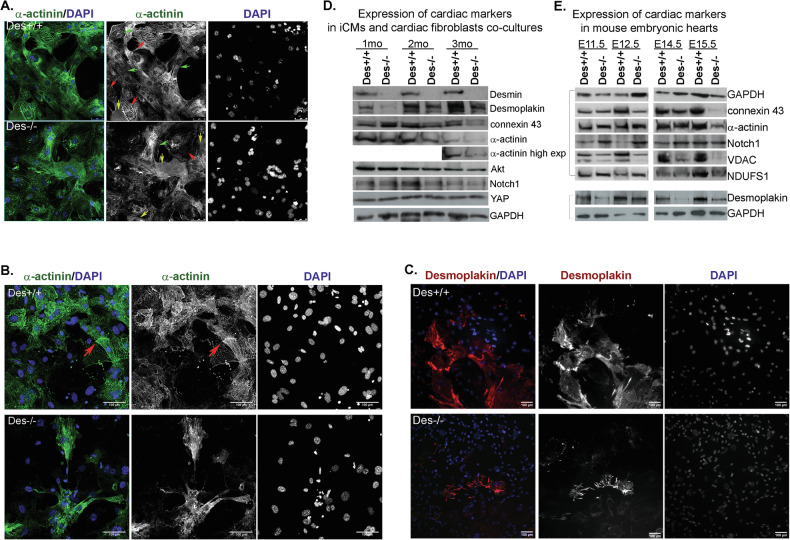
Desmin deficiency results in defects in the proper expression and localization of the cardiac markers α-actinin and desmoplakin during iCMs maturation and cardiac embryonic development. **A** Immunofluorescence staining for α-actinin in iCM^*Des+/+*^ and iCM^*Des−/−*^ cultures fixed 21 days after GMT transduction (red arrows indicating ‘high’ α-actinin expression, green arrows indicating ‘medium’ α-actinin expression, yellow arrows indicating ‘low’ α-actinin expression). *n* = 3. Green staining: α-actinin. DAPI: blue staining of nuclei. Scale bars: 50 μm. **B** Immunofluorescence staining for α-actinin in iCM^*Des+/+*^ and iCM^*Des−/−*^ cultures fixed 5 weeks after GMT transduction. *n* = 3, (red arrows indicate intercalated disks, IDs). Green staining: a-actinin. DAPI: blue staining of nuclei. Scale bars: 100 μm. **C** Immunofluorescence staining for desmoplakin in iCM^*Des+/+*^ and iCM^*Des−/−*^ cultures fixed 14 weeks after GMT transduction. *n* = 3, Scale bars: 100 μm. **D** Western blot analysis of whole-cell lysates from co-cultures of iCMs+CF grown up to 1-month, 2-months and 3-months after GMT transduction. *n* = 3. **E** Western blot analysis of whole-cell lysates from cardiac tissue of embryonic days E11.5, E12.5, E14.5 and E15.5. *n* = 3 (Two biological replicates are shown with the corresponding housekeeping protein for normalization.). iCMs induced cardiomyocytes.

In addition to examining the role of desmin in early cardiac cell differentiation, we investigated its importance in cardiomyocyte maturation during later stages of the trans-differentiation process. We examined the expression and localization of the cardiac markers, Α-actinin, desmoplakin and connexin 43, in the presence or absence of desmin (Fig. 4 and Fig. S3). FACS-isolated iCMs transduced with the GMT cocktail and cultured for 21 days were analyzed by immunolabeling. α-actinin expression defined three iCM categories based on expression and localization: “low” (diffuse, low-level expression), “medium” (medium-level expression and organized speckles), and “high” (high-level expression and sarcomere-like organization, indicating advanced maturity [36]). Significant differences were observed between *Des*^*+/+*^ and *Des*^*−/−*^ iCMs. iCMs^*Des+/+*^ showed a higher proportion in the “medium” and “high” categories (35.3% and 21.7%) compared to iCMs^*Des−/−*^ (25.95% and 16.43%), with 30% fewer in the “low” category (16.5% vs. 22%). (Fig. 4A and Fig. S3A). In further analysis, 2 months after transduction, iCMs^*Des+/+*^ displayed higher levels of α-actinin localized at well-defined *Z-discs*, forming intercalated disc-like structures (IDs), absent in *Des−/−* cultures (Fig. 4B, red arrowheads). IDs are the membrane structures that couple cardiac cells mechanically, electrically and chemically to function in coordinated syncytia.

Desmoplakin, a desmin interactor, localized at the desmosomes of IDs, and connexin 43, localized at gap junctions of IDs, followed similar patterns. There was a drastic decrease in the abundance of connexin 43 (almost 10-fold) and desmoplakin (3-fold) in the absence of desmin, with the subcellular distribution of the latter at IDs being disorganized (Fig. 4C and Fig. S3B, C, D).

Immunoblot analysis of iCMs co-cultured with cardiac fibroblasts for an additional 1–3 months confirmed significant downregulation of α-actinin, desmoplakin, and connexin 43 in iCMs^*Des−/−*^, particularly at later stages post-GMT transduction (Fig. 4D and S3Ei). Notch1, Akt1, and YAP protein levels did not differ between genotypes at these late stages, contrasting with earlier time points, attesting to a temporal role for desmin in Notch pathway regulation with potential compensatory mechanisms during prolonged maturation (Fig. 4D and S3Ei).

To determine the in vivo relevance of our findings, we isolated mouse embryos from embryonic day E11.5 to E15.5 when the heart has already undergone its major reorganization, but trabeculation is still in progress [48]. Protein profiling confirmed that iCM cultures faithfully reflected in vivo alterations (Fig. 4E). In *Des*^*−/−*^ embryos, cardiomyocyte maturity markers were markedly reduced from E11.5 onward, with greater deficits by E15.5. Desmoplakin decreased by 20–30% at E11.5 and >60% at E15.5; connexin43 declined by 50% at E12.5 and >70% at E15.5; α-actinin showed a 10% reduction at E12.5, rising to 30% at E15.5, relative to *Des*^*+/+*^ hearts. Additionally, voltage-dependent anion channel (VDAC)—a multifunctional mitochondrial protein and known desmin interactor—was diminished in *Des*^*−/−*^ embryos, consistent with reductions observed in adult *Des*^*−/−*^ hearts [49] (Fig. 4E and Fig. S3Eii).

These findings highlight desmin’s role in regulating proteins essential for signal transduction and cell–cell communication. Thus, we examined its impact on cardiomyocyte network formation in our in vitro culture system of iCMs^*Des+/+*^+ CFs and iCMs^*Des−/−*^+CFs. Brightfield microscopic analysis showed a progressive clustering of cells within the first four weeks after GMT transduction, forming “niches” predominantly composed of iCMs, as confirmed by GFP fluorescence, with occasional non-transduced CFs (Fig. S4B). By 12 weeks, these clusters elongated and interconnected (Fig. S4A). While the networks formed by iCMs^*Des+/+*^ exhibited complex, multiple “branches” that extended throughout the culture surface (Fig. S4Ai), the iCMs^*Des−/−*^ networks were markedly shorter, less branched (Fig. S4Aii) and 2.5-fold reduced in length (Fig. S4C). These findings strongly demonstrate that desmin is essential for proper cardiomyocyte connections, possibly implying an effect on intra- and intercellular signaling.

**Fig. 5:**
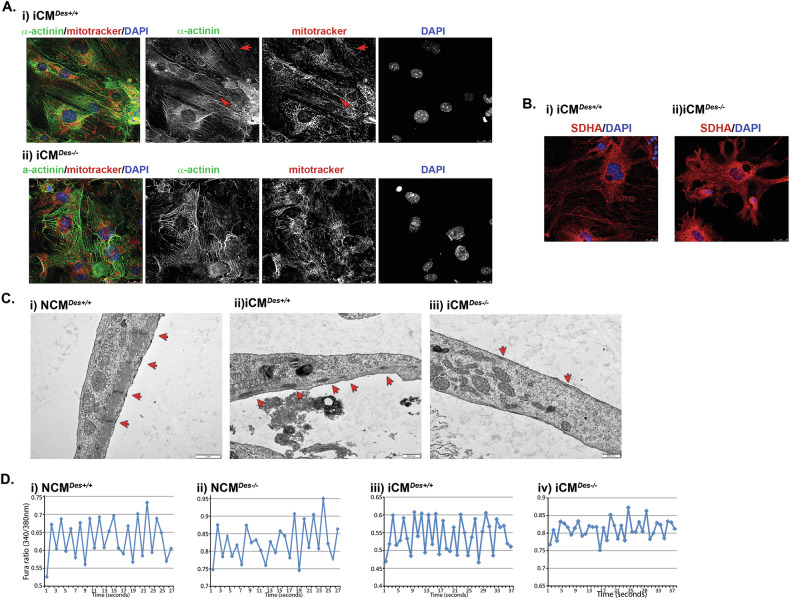
Desmin deficiency in iCMs leads to major delays in myofibrillogenesis and defects in Ca^2+^ influx. **A**, **B** Confocal microscopy images of (**A**) mitotracker red (red) and α-actinin (green) on i) iCMs^*Des+/+*^ and ii) iCMs^*Des−/−*^, 5 weeks after GMT transduction: red arrows show co-alignment of mitotracker with α-actinin. There is a better alignment of the mitochondrial network with Z-lines in the presence of desmin. **B** Mitochondrial staining with the mitochondrial matrix protein SDHA (red) on iCMs^*Des+/+*^ and iCMs^*Des−/−*^, 5 weeks after GMT transduction, shows a diffused and fragmented mitochondrial network when desmin is absent. DAPI: blue staining of nuclei. Scale bars: 25 μm. **C** Representative electron microscopy images of i) neonatal cardiomyocytes of *Des*^*+/+*^ (NCM^*Des+/+*^), ii) iCM^*Des+/+*^ and iii) iCM^*Des−/−*^: red arrows point to z-discs of assembled myofibrils, obvious in iCM^*Des+/+*^ and absent in iCM^*Des−/−*^. While more than 90% of the iCM^*Des+/+*^ cultures show myofibril formation, less than 10% of the iCM^*Des−/−*^ ones show any. Scale bars: 500 nm. **D** Representative calcium transient traces from the indicated cell types depicted as Fura-2-AM ratios (340/380 nm).

Given the established role of desmin in mitochondrial positioning and biogenesis in adult cardiomyocytes [2, 49], its influence on mitochondrial organization was assessed during cardiomyocyte development. Mitotracker staining (Fig. 5A) 5 weeks post-transduction revealed no major differences between the mitochondrial networks of iCMs^*Des+/+*^ and iCMs^*Des−/−*^. However, mitochondria in iCMs^*Des+/+*^ displayed a better alignment along the sarcomere when compared to the iCMs^*Des−/−*^, indicative of maturation (yellow staining in Fig. 5A). At D21, SDHA immunostaining demonstrated a well-defined mitochondrial network in iCMs^*Des+/+*^, contrasting with the fragmented and diffused signal in iCMs^*Des−/−*^ (Fig. 5B), suggesting immature mitochondrial architecture.

Electron microscopy after 3 months post-transduction revealed myofibrils with clear Z-disc structures in iCMs^*Des+/+*^, resembling those present in neonatal cardiomyocytes. Z-discs mark the boundaries of each sarcomere, the basal contractile unit of the myocyte. In contrast, iCMs^*Des−/−*^ exhibit minimal myofibril formation and lacked discernible Z-discs (Fig. 5C, red arrows).

All the above strongly suggest insufficient cardiomyocyte function. Indeed, intracellular Ca²⁺ analysis revealed markedly reduced and irregular transients in iCMs^*Des−/−*^ compared to the transients in iCMs^*Des+/+*^ at five weeks post-transduction, indicating impaired cardiomyocyte function (Fig. 5Diii, 5Div). Similar abnormalities were observed in neonatal cardiomyocytes (NCMs) from *Des*^*−/−*^ mice compared to *Des*^*+/+*^ (NCM^*Des−/−*^ vs. NCM^*Des+/+*^)(Fig. 5Dii, 5Di), demonstrating a cardiac defect present from early life and persisting into adulthood. Notably, iCMs^*Des+/+*^ displayed Ca²⁺ profiles comparable to NCM^*Des+/+*^, whereas iCMs^*Des−/−*^ exhibited even greater irregularity than NCM^*Des−/−*^. These findings demonstrated that reprogrammed iCMs accurately predict the in vivo picture of cardiac maturity and function.

### Desmin affects global RNA expression of cardiac-specific modulatοrs and chromatin architecture from early development to adulthood

Mechanical forces are crucial modulators of cellular processes, including gene expression. The desmin cytoskeleton, through its association with the lamin A/C nucleoskeleton, likely functions as a mechanosensor, forming a dynamic structure that integrates and regulates inter- and intracellular signaling, stress responses and chromatin organization, thereby influencing nuclear events. Electron microscopy revealed that nuclei from desmin-expressing cardiomyocytes were elongated with smooth nuclear membranes, whereas those from desmin-deficient cardiomyocytes were shrunken and displayed irregular lamina morphology with recesses and projections (Fig. S5), indicating altered nuclear architecture.

**Fig. 6:**
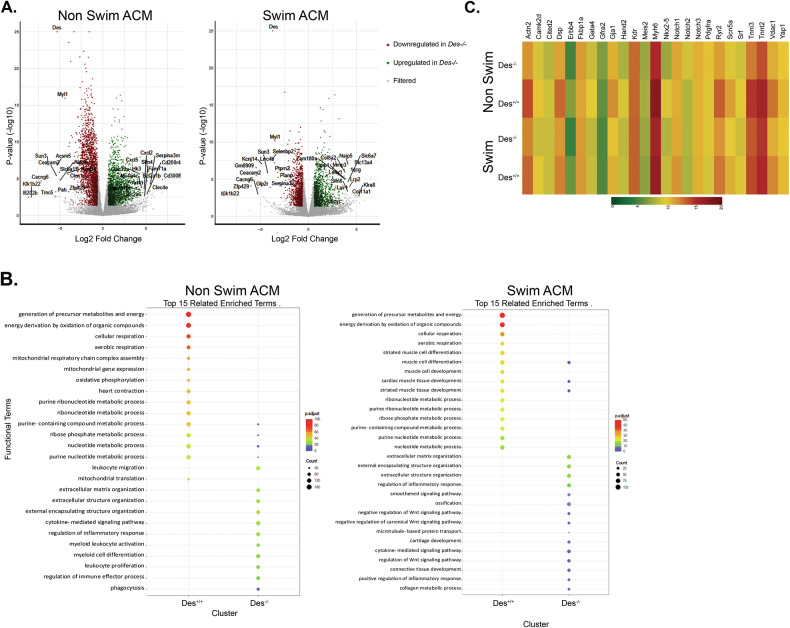
Desmin deficiency results in large-scale changes in gene expression. **A** Volcano plot displaying differentially expressed genes as identified by RNA sequencing performed in adult cardiomyocytes (ACM) isolated from *Des*^*+/+*^ and *Des*^*−/−*^ 3-month-old mice under basal and swimming stress conditions. Significantly different genes were considered with a log2FC cutoff of **≥** |0.6 | , *p*-value ≤ 0.05 and counts ≥20 (**B**) GO annotation analysis showed a significantly altered group of genes in the absence of desmin. **C** Heatmaps showing expression change of representative genes important for cardiogenicity and also identified in the RNA seq performed on D7 iCMs (Fig. 1). ACM adult cardiomyocytes, *p*-adjust: adjusted *p*-value = negative log of 10, counts: number of genes in each category.

To directly explore the global transcriptional impact of desmin, RNA sequence analysis was performed on adult cardiomyocytes from 3-month-old mice with or without swimming-induced stress (Fig. 6). The absence of desmin influences the proper expression levels of a high number of genes under basal or stress conditions. In the *Des*^*−/−*^ background under basal conditions, 2094 genes are upregulated and 2138 are downregulated, while under swimming stress conditions, 1319 genes are upregulated and 1122 are down (Fig. 6A). Gene Ontology (GO) analysis revealed that, compared to the *Des*^*−/−*^ background, the *Des*^*+/+*^cardiomyocytes showed an enrichment in genes involved in metabolism and mitochondrial function under both basal and stress conditions. Conversely, genes upregulated in *Des*^*−/−*^ cardiomyocytes were primarily linked to inflammatory response. Under stress conditions, desmin is required for the adequate expression of genes involved in cardiac processes such as cardiac cell development and differentiation, contractility, and for the positive regulation of the signaling pathways that control cardiac development and repair (Fig. 6B). This corroborates our results, which show that desmin is necessary for proper expression of cardiac-specific regulators. Notably, key regulators identified in earlier RNA analysis (Notch1, Notch2, Notch3 and ErbB4), presented a similar pattern throughout development and in adulthood (Fig. 6C). This data suggests that desmin consistently regulates overlapping gene networks both in adulthood and early in development.

**Fig. 7:**
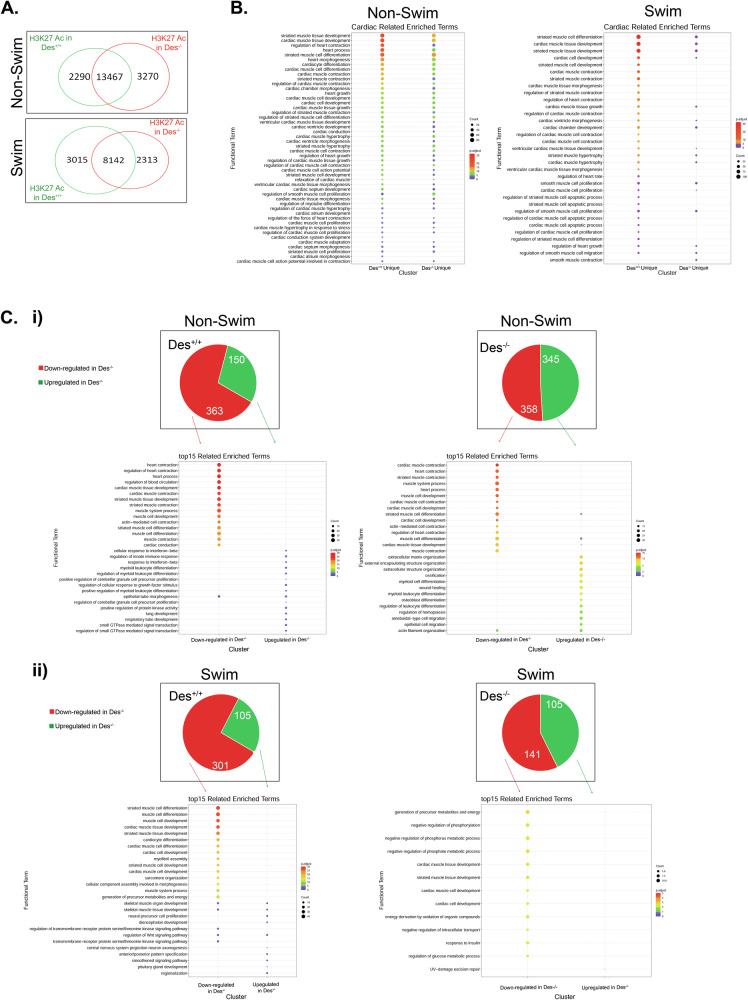
Desmin influences active chromatin modification status and enhances the expression of genes involved in cardiac processes. **A** Venn diagram displaying unique and common peaks found by H3K27Ac ChIP on the *Des*^*+/+*^ and *Des*^*−/−*^ backgrounds under basal (top diagram) and swimming (bottom diagram) conditions. **B** GO annotation analysis showing the most significantly altered groups of genes. **C** Correlation of the RNA and ChIP analysis on ACM isolated from *Des*^*+/+*^ and *Des*^*−/−*^ mice under basal and swimming stress conditions. (**Ci**) Pie charts showing differentially expressed genes found in RNA sequence in *Des*^*+/+*^ background (left top panel) and *Des*^*−/−*^ background (right top panel) from the unique peaks found in ChIP seq comparing the two backgrounds. Bottom panels showing the GO annotation of the groups of these altered genes. (**Cii**) Similar to (Ci) under stress conditions. H3K27Ac ChIP: chromatin immunoprecipitation against Histone H3, Lysine 27 acetylation. *p*-adjust: adjust *p*-value = negative log of 10, counts: number of genes in each category.

To unravel the molecular basis of the observed desmin-dependent transcriptional differences, we examined histone modification patterns in the *Des*^*+/+*^ and *Des*^*−/−*^ backgrounds. Among multiple lysine acetylation sites, H3K27 Ac serves as a robust marker for active enhancers [50]. ChIP sequence profiling of H3K27 Ac in adult cardiomyocytes revealed distinct active chromatin landscapes (Fig.7A). Under basal conditions, *Des*^*+/+*^ cardiomyocytes displayed 2290 unique peaks, *Des*^*−/−*^ 3290, and there were 13467 common peaks. Under swimming stress, the respective numbers were 3015, 2313 and 8142 (Fig. 7A). GO analysis revealed that *Des*^*+/+*^ unique peaks were associated with genes significantly involved in cardiac-related processes, such as cardiac cell differentiation, development, growth, contraction and apoptosis, whereas *Des*^*−/−*^ unique peaks lacked this involvement (Fig. 7B left panel). These differences were more pronounced under stress (Fig. 7B right panel).

Integration of the above RNA-seq and ChIP-seq datasets revealed that in the *Des*^*+/+*^ environment, unique acetylated peaks associated with upregulated expression of genes encoding cardiac-related proteins, while downregulated genes were largely unrelated to cardiac function (Fig. 7Ci, Cii left panel). Conversely, in the *Des*^*−/−*^ background, the unique peaks were linked to upregulated expression of genes unrelated to cardiac processes, while the downregulated ones were cardiac-related. (Fig. 7Ci, Cii right panel). This pattern persisted and intensified under stress (Fig. 7Cii). Even the common peaks exhibited higher cardiac-related gene expression in *Des*^*+/+*^ than in *Des*^*−/−*^ (Fig. S6). Collectively, these findings indicate that desmin promotes histone acetylation profiles favoring cardiac gene activation, thereby coupling structural integrity to transcriptional programs essential for cardiomyocyte function.

Given desmin’s proposed role in chromatin organization via its interaction with nuclear lamins through the LINC complex, we performed Lamin A/C ChIP-seq. Lamin A/C is the major nuclear intermediate filament of the cardiomyocyte. Despite low read counts, results paralleled H3K27Ac data. Post-stress, *Des*^*+/+*^ unique peaks associated with cardiac differentiation/development genes, whereas this association was attenuated in *Des*^*−/−*^, suggesting impaired lamin–chromatin interactions without desmin. (Fig. S7).

**Fig. 8:**
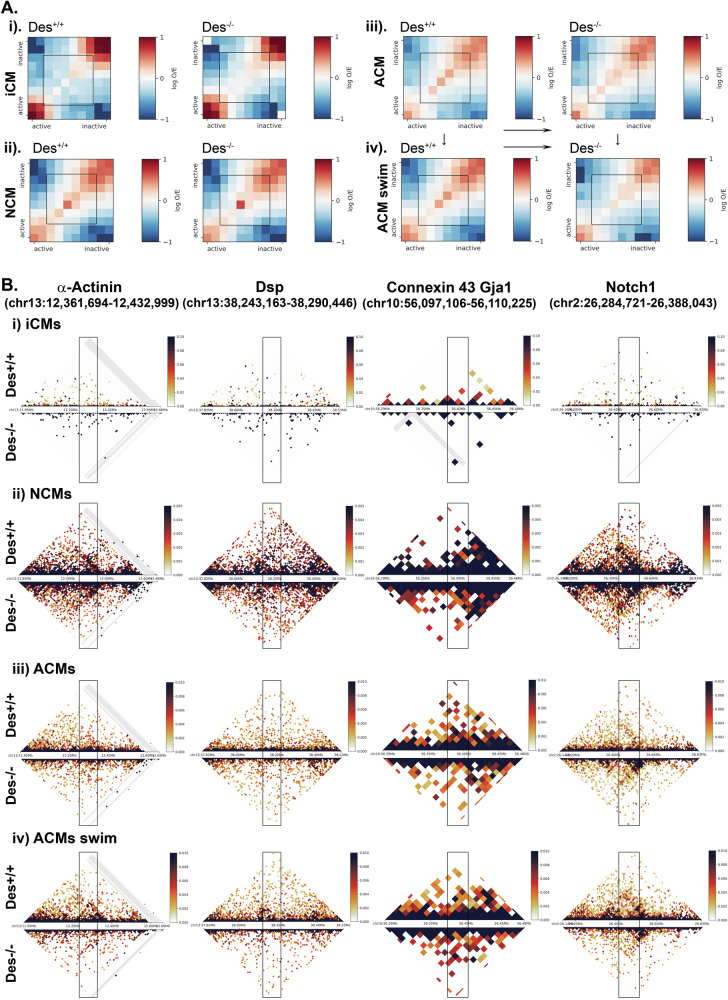
Desmin affects active and inactive chromatin compartments of cardiomyocytes at a megabase scale. **A** A/B compartment heatmaps after FAN-C analysis of cardiomyocyte genomes from *Des*^*+/+*^ and *Des*^*−/−*^ backgrounds at different time points. (Ai) on iCMs: early in trans-differentiation at D7 (condition ‘a’), (Aii) on NCM, at day 1-3 post birth (neonatal) (condition ‘b’), (Aiii) on ACM isolated from 3month old mice (adulthood) (condition ‘c’) and (Aiv) on ACM swim: isolated from 3month old mice undergone a swimming exercise stress (condition ‘d’). Resolution 1 mb. **B** Representative of FAN-C analysis at 10Kb resolution of the genome contact matrix at the conditions mentioned in (**A**) on specific chromosomes where genes of interest are located. Shown: α-actinin and desmoplakin (chr13:38,151,294-38,198,577 and chr13:12,269,426-12,340,760), Connexin 43 or Gja1 (chr10:56,388,423-56,388,482), Notch1 (chr2:26,459,531-26,503,558). Long light blue boxes cover the locations of the specific genes. The color maps represent relative chromatin interaction probability and are displayed on the same scale for each condition for comparison. iCMs induced cardiomyocytes, NCMs neonatal cardiomyocytes, ACMs adult cardiomyocytes, Genome browser (https://genome.ucsc.edu) was used for gene locations.

To access further desmin’s influence on chromatin architecture, we performed a Hi-C (chromosome conformation capture [51]) analysis across 4 different conditions critical for cardiac function: a) D7 iCMs^*Des+/+*^ and iCMs^*Des−/−*^ to determine chromatin early differentiation-associated differences; b) neonatal cardiomyocytes (NCM) from *Des*^*+/+*^ and *Des*^*−/−*^ mice at day 1–3 (D1-3) after birth, when morphology and function appear similar; c) adult cardiomyocytes (ACM) from 3 month-old *Des*^*+/+*^ and *Des*^*−/−*^ mice, a time point where the pathology of dilated cardiomyopathy has developed in the *Des*^*−/−*^ mouse model and d) ACM from the 3-month mice, after swimming exercise stress. Global chromatin architecture features, including topologically associated domains (TADs), showed no major differences. However, examination of active and inactive chromatin compartments (arbitrarily called A and B compartments respectively) revealed genotype-specific differences (Fig. 8A), most prominent pronounced in the D7 iCMs (condition ‘a’) and the post-exercise ACMs (condition ‘d’). In all conditions, same-type compartment contacts (A-A, B-B) were more frequent than different-type compartment contacts (A-B). A reduction of A-B interactions was observed in conditions ‘c’ and ‘d’ in the ACM^*Des−/−*^, basal and swim, compared to their *Des*^*+/+*^ counterparts, whereas a slight increase of A-B interactions was observed in iCM^*Des−/−*^ and NCM^*Des−/−*^ compared to their *Des*^*+/+*^ counterparts. (Fig. 8A).

Subsequently, we examined specific loci to determine how desmin affects the local interactome and organization (Fig. 8B). Specifically, the *Notch1* locus displayed different chromatin arrangement across all conditions, consistent with its altered transcription upon desmin deficiency. Notably, multiple regulatory elements for *Notch1* lie within its gene body (Fig. S8), suggesting that proximal structural changes could strongly affect its expression. The *α-actinin* locus also exhibited genotype-specific patterns, particularly in adult cardiomyocytes (condition ‘c’). Likewise, in ACMs under basal and stress conditions (‘c’ and ‘d’), conformational changes were observed on chromosome 13, proximal to where *connexin 43* and *desmoplakin* genes are located. NCMs (condition ‘b’) showed minimal alteration in the regions analyzed.

Further analysis of desmin-affected genes, including *Notch2* and those implicated in CPC and maturation studies (i.e., *Pdgfra*, *Kdr*, *VDAC*) (Fig. S9A), showed similar interactome differences. The chromatin neighborhood of *Notch2* in early differentiation (condition ‘a’) differed between *Des+/+* and *Des−/−* conditions (Fig. S9Ai), paralleling RNA-seq findings at both early differentiation and later adulthood. Differences were also detected for the *VDAC* mitochondrial gene neighborhood. There were undetectable HiC changes on loci encoding cardiac progenitor markers under any condition.

The above data support the hypothesis that desmin plays a dual role in cardiac-specific expression, an early one impacting significantly cell differentiation and development and a later one in homeostasis, especially under stress conditions.

## Discussion

This study provides the first comprehensive assessment of desmin’s spatiotemporal role in early cardiac differentiation, as well as postnatal and adult stages. Using the direct trans-differentiation approach of fibroblasts to iCMs, we explored desmin’s contribution in cardiogenesis. iCMs resemble neonatal cardiomyocytes and model their maturity more closely than iPSC-induced cardiomyocytes (iPSC-CMs) [52] do. Direct reprogramming opens doors to new personalized regenerative approaches, which, unfortunately, remain limited due to the gaps in understanding the molecular determinants of lineage commitment and differentiation.

Our results demonstrate that desmin modulates cardiac differentiation at multiple levels, including lineage determination, cell differentiation, and tissue homeostasis. We showed that desmin enhances the initial steps of differentiation, since its ectopic expression increased the iCM reprogramming efficacy of the GMT cocktail. While previous work implicated desmin in myogenesis and cardiogenesis by regulating factors, such as Mef2C, Brachyury, Goosecoid, and Nkx2.5 [20–24], this is the first report showing its role in promoting CM-like fate through transcriptional regulation of key proteins and signaling pathways. Our transcriptomic profiling indicates that desmin affects *Notch1* expression in a spatiotemporal manner, activating the pathway during the early differentiation process and then in adulthood under stress, while its depletion impaired it. These findings align with Notch1’s known developmental and stress-responsive function. It is documented that Notch1 is activated in proliferating embryonic and immature cardiomyocytes, whereas its expression is downregulated in the postnatal myocardium. Later, in the adult myocardium, Notch1 is transiently reactivated following myocardial stress or injury, suggesting a potential protective role for Notch signaling in response to cardiac stress and damage [38, 53–57]. Additionally, defective regulation of the HIPPO/YAP shown in *Des*^*−/−*^ mice suggests that desmin plays a significant role in cardiac regeneration, consistent with our more recent findings concerning the side population (SP) of adult cardiac stem cells [30], a hypothesis that requires further examination.

RNA-seq analysis of D7 iCMs revealed desmin-dependent regulation of additional Notch pathway members, such as *Notch2*, *Fkbp1a*, and *Mib1,* which are key mediators of cardiac trabeculation [41, 48], a process essential for pre-vascular blood oxygenation and ventricular conduction system development. Improper trabeculation leads to embryonic lethality or congenital cardiomyopathy [48]. We showed that *Des*^*−/−*^ embryonic hearts exhibited ventricular hypertrabeculation, resembling, though to a lesser degree, Notch pathway-related defects, underlying Ventricular Non-Compaction Cardiomyopathy (VNCC), a congenital heart disease (CHD) [38]. Notably, human desmin mutations have been linked to VNCC [58, 59], and we have shown that it influences other CHD-associated markers, such as Nkx2.5 [30, 31], supporting a role in congenital heart disease pathogenesis.

Furthermore, we showed that desmin is crucial for the maintenance of cardiac progenitors *PDGFRa*^*+*^/*GFRa2*^*+*^*/Kdr*^*+/low*^ CPCs as early as E7.5, explaining the observed developmental defects in heart embryos and establishing itself as a potential CPC marker.

Our in vitro and in vivo studies indicate that desmin deficiency delays cardiac-specific protein expression and overall maturation, making it a good target for regenerative approaches, since the immature phenotype of laboratory-generated cardiomyocytes remains an obstacle to effective regeneration therapies. Compromised levels and mislocalization of α-actinin, desmoplakin and connexin 43 strongly indicate that desmin is essential for proper expression, trafficking, and localization of intercalated disc (ID) proteins, thereby ensuring functional communication between cardiomyocytes and between cardiomyocytes and non-cardiomyocytes. The observed molecular and cellular defects in iCMs^*Des−/−*^ led to a delay in myofibril assembly and myofiber formation, resulting in structural disorganization and impaired excitability and contractility, as confirmed by irregular Ca²⁺ signaling. While previous studies identified desmin-related cardiac defects manifesting in adulthood [13], causing dilated cardiomyopathy, our in vivo data from iCMs and neonatal cardiomyocytes reveal that these defects originate early upon development.

To identify the mechanism with which desmin performs its multiple roles, we further explored the link between the desmin IF network and nuclear organization. Loss of desmin results in nuclear deformation of skeletal and cardiac muscle [49, 60–62], and our studies showed that in desmin knockout cardiomyocytes, the nucleus appears to be infolded or fragmented. These deformities are consistent with the “push-pull balance” model of IF-microtubule regulation of nuclear homeostasis [63]. Moreover, previous observations of desmin transiently entering the nucleus of ESC-derived CPCs [31] suggest a role in chromatin regulation. Our RNA and ChIP sequencing on adult cardiomyocytes answered a long-standing hypothesis. These findings affirmed the essential role of desmin in gene regulation programs controlling cardiac function, metabolism, tissue development, cell differentiation and maturation, with pronounced effects under stress. Importantly, the same pathways were pinpointed also in D7 iCMs, depicting changes at the early steps of differentiation in a non-pathological environment, thus excluding from our evaluation any secondary effects and confirming a direct role of desmin in the transcriptional control of cardiogenesis, development and long-term homeostasis.

Hi-C analysis revealed that the loss of desmin significantly altered the chromatin landscape, with the severity of the alterations depending on environmental conditions during both development and adulthood. During embryogenesis, desmin appears to be essential for proper modulation of mechanotransduction, including buffering stress signals that influence development, while in adulthood, this additional role remains critical in adapting to stressors associated with greater pressure and volume overload. The integrity of the desmin IF network was therefore indispensable for proper cardiac stress responses across developmental stages.

Direct reprogramming highlighted the importance of this three-dimensional cytoskeletal network in cardiomyocyte formation and maturation. Desmin ensures proper myocyte homeostasis both within a single myocyte as well as throughout the entire muscle tissue, thereby serving as a fundamental regulator of cardiac maturation and maintenance.

It is of great importance to realize that the IF cytoskeleton plays a key role in the integration of structure and function through mechanotransduction both during embryonic development and in adulthood. In adult cardiac and skeletal muscle, the IF cytoskeleton associates with the contractile apparatus, membranes and membranous organelles, such as mitochondria and nuclei, thereby facilitating the integration and coordination of growth and energy demands of the working myocyte (extensively reviewed in [2, 4, 5, 9, 12]). This coordination relies not only on mechanical force transmission, but also de novo gene expression, energy production, protein and lipid trafficking and targeting, particularly through communication of mitochondria with nuclei and other organelles. While initially recognized in skeletal muscle during exercise, these processes are now evident to be critical also in the heart, which experiences constant exercise-like stress, both in adults and developing embryos. As extensively described, failure of this coordination due to mutations in the IF desmin-lamin or associated proteins leads to mitochondrial and other organelle defects, cell death and heart disease [2, 4, 5, 13–19, 49]. As such, desmin arises as a prime example that the role of IFs in the coordination of cellular processes necessary for muscle-specific cell determination, differentiation, growth, development and health maintenance extends far beyond traditional thinking.

## Methods and materials

### Generation of αMHC-GFP/Desmin^+/+^ and αMHC-GFP/Desmin^−/−^ mice

αMHC-GFP mice were obtained from Srivastava laboratory [34] and were backcrossed five times with a wild-type mouse background 129SV to generate *αMHC*^*GFP/GFP*^*/Desmin*^*+/+*^ (*Des*^*+/+*^, for simplicity). These mice were then crossed with *Desmin*^***−/−***^ of 129SV background to generate *αMHC*^*GFP/GFP*^*/Desmin*^*−/−*^ mice (*Des*^*−/−*^ for simplicity). All animal procedures were performed in accordance with the guidelines for animal experimentation of the Committee for evaluation of animal protocols and the monitoring Committee on the animal well-being of the animal facility of the Biomedical Research Foundation of Academy of Athens (BRFAA) institute (Auth. Number #6957/2016) following the guidelines of the Greek Ministry of Agricultural Development and Food Administration and the Directorate of Veterinary and Sustainable Animal Production. All the above conformed to the Guidelines of the Association for the Assessment and Accreditation of Laboratory Animal Care (AAALAC) and the recommendations of the Federation of European Laboratory Animal Science Associations (FELASA). The animals were used independent of sex. The procedures are designed to minimize animal suffering and respect the 3R principles. For euthanasia, mice were anesthetized with inhaled 5% isoflurane for several minutes; then, anesthesia was confirmed via tail pinch, and cervical dislocation was performed before chest incision and heart removal. The number of animals for each group was calculated according to G power analysis *t*-test: using values for effect size 0,8, power = 0.80 and significance level = 0.05. All data were analyzed with analysis of variance (ANOVA) and/or unpaired Student’s *t*-test where appropriate. A value of *p* < 0.05 was considered significant.

### Embryo isolation and cardiac tissue dissection

Pregnant females were euthanized at defined gestational time points (E7.5, E8.5, E9.5, and E10.5) to isolate embryos. For E7.5 embryos, the posterior region containing the cardiac crescent was carefully dissected, while extraembryonic structures, including the ectoplacental cone and allantois, were removed. The cardiac crescent and developing heart tissue were enzymatically dissociated using a digestion solution containing Collagenase D (2 mg/mL), Trypsin (1 mg/mL), and DNase I (1 mg/mL). Digestion was carried out at 37 °C for 30 min to facilitate single-cell suspension or further downstream.

### Cell culture

Cardiac fibroblasts (CFs) and neonatal cardiomyocytes from 1–3-day-old neonatal mice and adult cardiomyocytes (ACM) from 3-month-old mice were isolated as described previously with minor modification [35, 49, 64].

### Molecular cloning, generation of retroviruses and retroviral infection

Retroviral transduction and cellular reprogramming were performed as described [34, 35, 64].

Retroviral vectors (pMXs-GATA4, pMXs-Mef2C, pMXs-TBX5) were obtained from the Srivastava lab [34]. Full-length desmin cDNA was cloned into the pMXs vector, and DsRed from the pDsRed2-N1 vector was cloned into pMXs as a control.

Platinum E cells (Retroviral Packaging Cell Line, Ecotropic (Plat-E), CELL BIOLABS, RV-101) were grown according to the manufacturer's protocols.

For the generation of retroviruses, eight micrograms of retroviral plasmid DNA was transfected using PEI (Polyethylenimine, linear (Polysciences)) into Platinum E cells, which were plated at 70% confluency 24 h prior to transfection. Sixteen hours after transfection, the medium was changed to fresh medium (DMEM/M199 (4:1) with 10% FBS and antibiotics). After 36 h of transfection, viral medium was harvested every 8 h for three times (total 24 h) and filtered through a 0.45 μm cellulose filter. The viral supernatant was mixed with polybrene (Sigma) to a final concentration of 6 μg/mL.

Retroviral infection CFs, from 1–3-day old neonatal mice, was performed as previously described [34, 64] using a viral mixture of DGMT or RGMAT in a ratio 2:1:1:1, and of GMT in a ratio 1:1:1.

### FACS (fluorescence-activated cell sorting) analyses and sorting

For GFP expression analyses, transduced cells were harvested and analyzed on a FACS ARIATM II (BD Biosciences) at different time points after transduction. GFP-positive sorted cells were plated on collagen-A-treated plates and analyzed at different time points.

For the analysis of desmin expression in the CPC population, endogenous GFP and CPC markers in embryonic cardiac cells were measured after cardiac crescent/ heart isolation of embryonic day E7.5, E8.5, E9.5 and E10.5. Cells were fixed with 3.7% formaldehyde and stained with desmin (AbCam ab185033), Kdr (Biolegend 121910), PDGFRa (AbCam ab93531), GFRa2(R&D AF429) antibodies and DAPI (Abcam ab175664). Flow cytometry was performed with the Aria™ II machine BD and analyzed with FlowJo software.

### RT-qPCR and western blot analyses, histology, immunocytochemistry, and electron microscopy

Total RNA was isolated from cells; cDNA was synthesized by reverse transcription, and qRT-PCR was performed on an ABI 7900HT (Applied Biosystems) using SYBR green (Roche) technology. Primer information is available on request. mRNA levels were normalized to β-actin mRNA.

Western blot analyses were performed using the following antibodies, anti-: α-actinin (Sigma 1:1000), desmoplakin (Invitrogen-ThermoFisher 1:500), desmin (Santa Cruz 1:1000), connexin43 (Sigma 1:2000), Notch1 (Cell Signaling 1:1000), YAP (novus 1:1000 and Cell Signaling 1:1000), Akt1 (Cell Signaling 1:1000), GAPDH (Invitrogen 1:3000), b-tubulin (Cell Signaling 1:1000), Lamin A/C (Santa Cruz 1:1000), VDAC (Cell Signaling 1:1000). For the protein level changes in the maturity studies of the iCMs, a co-culture of cardiac fibroblasts and transduced iCMs were harvested with Laemmli sample buffer at different time points after transduction. For heart lysates, hearts were isolated from mice of different ages, homogenized in nuclear extraction buffer (10 mM HEPES pH 7.9, 0.42 M KCl, 0.1 mM EDTA, 0.1% NP40, 10% glycerol, 1 mM DTT and protease inhibitors) with a Dounce homogenizer and the protein was quantified by Bradford. For cell fractionation, hearts were isolated from 3-month-old mice and homogenized by Dounce homogenizer in hypotonic buffer (20 mM HEPES, pH 7.9, 7 mM KCl, 1.5 mM MgCl, 0.1 mM EDTA, 1 mM DTT and protease inhibitors). The homogenized sample was centrifuged at 500 x *g* for 10min. The nuclear pellet was resuspended in nuclear buffer.

For histology, isolated embryos/hearts were fixed in 10% formalin for 16 h and then underwent tissue paraffin treatment. 5 μm sections were cut on a microtome, and hematoxylin/eosin staining was performed.

Measurements of trabeculae were performed using Fiji (ImageJ) software.

For immunofluoresence, cells were fixed in 4% paraformaldehyde for 15 min at RT, blocked with 5% BSA solution in 1x PBS and incubated overnight in the same solution containing primary antibodies: anti-GFP (Invitrogen 1:2000), anti-α-actinin (Sigma, 1:1000), anti-desmoplakin (Invitrogen-ThermoFisher 1:500), and anti-connexin43 (Sigma, 1:400). After washing with PBS/0.005% Tween, Alexa fluorogenic secondary antibodies (Molecular Probes-ThermoFisher Scientific 1:1500) were applied for 1 h at RT. Cells were washed with PBS/0.005% Tween, and nuclei were stained with DAPI. The samples were imaged by confocal microscopy (Leica SP5) using the appropriate laser line.

Measurements of signal intensity and area were performed using Fiji (ImageJ) software.

Electron microscopy was performed as described in [49].

### Ca^2+^ imaging

Mouse ventricular cardiomyocytes and iCMs were incubated in Tyrode’s buffer with 20 µM Fura 2-AM (Sigma) for 30 min at 37 ^o^C, washed, and incubated for an additional 30 min to allow de-esterification of the dye. Ratiometric Ca^2+^ measurements were performed for 200 sec with 2 s intervals at 340 and 380 nm using the PTI (Photon Technology International) Imaging System (Nikon TE 2000) with an automated fluorescence microscope and a CCD camera (PTI-IC200).

### Differential gene expression analysis (RNA sequencing)

RNA-seq experiments were carried out in the Greek Genome Center (GGC) of the Biomedical Research Foundation of the Academy of Athens (BRFAA). RNA was isolated with the Nucleospin RNA kit from Macherey-Nagel. RNA-seq libraries were prepared with the NEBNext Ultra II Directional RNA Library Prep Kit for Illumina, with 1 μg of total RNA input. For the first RNA-seq experiment (Fig. 1), DsRED+GMT and Desmin+GMT samples, one library of each sample was constructed from a pool of 6 independent experiments of iCM cells, which were isolated by Fluorescence-activated cell sorting flow cytometry. For the second RNA seq experiments, three (*n* = 3) libraries for each sample were constructed, each of which contained cardiomyocytes isolated from 3–4 different *Des*^*+/+*^ and *Des*^*−/−*^ 3-month-old mice. All libraries were checked with the Agilent bioanalyzer DNA1000 chip and quantified with the qubit HS spectrophotometric method Approximately 25 million, 100 bp long Single End Reads were generated per sample with the Illumina Novaseq 6000 sequencer.

QC of the raw sequencing reads was performed with the FASTQC and FastxToolkit software. Raw reads from RNA-Seq experiments were aligned to the GRCm38 (mm10) reference genome with the HISAT2 aligner with default options [65]. SAM files were converted to sorted and indexed BAM files using Samtools [66]. Counting reads to genes was done using the HTSeq—Count program with the options -s reverse-additional-attrgene_name [67]. Differentially expressed genes (DEGs) were identified using the DESeq2 R package [68]. DEG matrices from DESeq2 were filtered using the following criteria: log2 fold change >=0.6, *p*-value ≤ 0.05, and a mean count of ≥20 across all replicates in B condition or log2 fold-change <=−0.6, *p*-value ≤ 0.05, and a mean count of ≥20 across all replicates in A condition. Gene ontology analysis was performed using the clusterProfiler R package [69, 70].

### Chromatin immunoprecipitation (ChIP) and ChIP sequence data analysis

Adult cardiomyocytes isolated from *Des*^*+/+*^ and *Des*^*−/−*^ 3-month-old mice that have or have not undergone swimming stress exercise as previously described [49, 64]. H3K27Ac and Lamin A/C chromatin immunoprecipitation was performed on isolated chromatin from 5 mice of each genotype and each condition using 2 μg of antibody (H3K27Ac: Abcam ab4729 and Lamin A/C:Millipore MABT 1341) as previously described [71]. Input and IP DNAs were quantified with Qubit. 5–10 ng DNA from each sample was used as input for ChIP-seq library prep with the NEBNext Ultra II DNA library prep kit for Illumina, according to the manufacturer’s instructions. QC of the final purified libraries was performed with a Qubit HS and an Agilent Bioanalyzer DNA1000. Approximately 25 million, 100 bp-long single-end reads were generated per sample with the Illumina Novaseq 6000 sequencer. QC of the raw sequencing reads was performed with the FASTQC and FastxToolkit software. Raw reads from ChIP-Seq experiments were aligned to the GRCm38 (mm10) reference genome using the Bowtie2 aligner with default options [72]. SAM files were converted to sorted and indexed BAM files using Samtools [66]. Peak calling was performed using in-house scripts (https://github.com/supatt-lab/rChIPSeqTools/blob/master/sPeakDetection.R). Annotation of the peak lists was done using the ChIPseeker R package [73, 74]. Common and unique peak regions between ChIP-Seq experiments were computed using ChIPpeakAnno [75, 76]. Gene ontology analysis was performed using clusterProfiler [69, 74].

### Hi-C analysis

Hi-C was performed as described in Diaz et al. [51] and sequenced in Illumina Novaseq 6000 200cycles. To align the reads to the mm10 mouse genome version, as well as to perform the various downstream analyses, the FAN-C pipeline [77] was used. One replicate for each condition was run. The replicate of the iCM samples contained 1 × 10^6^ cells isolated by FACS sorting. There were six [6] separate FACS experiments in each condition (6 for the RGMT sample and 6 for the DGMT sample) performed, and cells were pooled together in order to collect the number of cells needed. For the NCM samples: 10^6^ pooled NCMs were collected from 3 different isolations of neonatal cardiomyocytes, each isolation included hearts from 10 pups, 1–3 days old. For the ACM samples, 10^6^ pooled ACMs were collected from 6 different 3-month-old mice of each genotype for each condition.

Hi-C data were analyzed using FAN-C. Raw reads for each pair were aligned to the mm10 mouse genome version and the generated sam files were sorted and used to generate filtered pair files, which were used to create Knight–Ruiz (KR) normalized multi-binned Hi-C matrices (5 mb, 2 mb, 1 mb, 500 kb, 250 kb, 100 kb, 50 kb, 25 kb, 10 kb, 5 kb) using FAN-C-auto with default parameters.

TAD boundaries were called using the insulation score method from FAN-C. Insulation scores were calculated for 10-kb binned HiC matrices at multiple window sizes (10 kb, 50 kb, 100 kb, 250 kb, 500 kb, 1 Mb, 1.5 Mb, 2 Mb, 2.5 Mb, 3 Mb, 3.5 Mb, 4 Mb). Domain boundaries were calculated from the insulation scores. Genome contact matrices were produced using 10 kb binned HiC matrices with a color scale of 0.1 for iCM, 0.03 for NCM and 0.01 for ACM and ACM swim.

A/B compartment heatmaps were produced using fanc compartments with eigenvector binning at 10, 20, 30, 40, 50, 60, 70, 80, 90, 100 using normalized 1 mb binned HiC matrices.

### Statistical analysis

All in vitro data are presented as mean ± SEM and have at least *n* = 3 biological replicates per group. Specific *n* values are indicated in each figure legend. *p*-values were calculated with Student’s two-tailed *t*-test and/or ANOVA. *p*-values are indicated in figure legends.

#### Ethics approval and consent to participate

This study was conducted in accordance with the principles outlined in the Declaration of Helsinki. Protocol and Ethics approval for this study was obtained from the following ethics committees, and it was given a protocol number/reference 6957/7-10-2016: 1. Hellenic Republic, Region of Attica—General Directorate of Agricultural Economy & Veterinary Affairs—Directorate of Agricultural & Veterinary Policy. 2. Ministry of Rural Development & Food—General Directorate of Sustainable Animal Production & Veterinary Affairs. 3. Directorate of Animal Protection, Pharmaceuticals and Veterinary Applications. 4. Directorate of Agricultural Economy & Veterinary Affairs, Central Sector of Athens Regional Unit. 5. Protocol Evaluation Committee of the Animal Standards Unit, Biomedical Research Foundation, Academy of Athens. 6. Animal Welfare Monitoring and Advisory Committee of the Animal Standards Unit, Biomedical Research Foundation, Academy of Athens. All participants involved in the study provided informed consent, and the study was performed in compliance with all applicable national regulations and guidelines. There were no human research participants involved in the study.

## Supplementary information

Supplemental figure legends

Supplemental Figure 1

Supplemental Figure 2

Supplemental Figure 3

Supplemental Figure 4

Supplemental Figure 5

Supplemental Figure 6

Supplemental Figure 7

Supplemental Figure 8

Supplemental Figure 9

Supplemental Table

Original western blots of Figures

## Data Availability

RNA-seq, ChIP seq, and HiC data have been deposited to GEO and are going to be publicly available after publication. GEO: GSE274613; Other original data reported in this paper will be shared by the lead contact upon request. This study does not report original code. Any additional information required to re-analyze the data reported in this paper is available from the lead contact upon request.

## Materials availability

Materials generated in this study are available through the lead contact upon request.

## Code availability

RNA-seq, ChIP-seq, and HiC data have been deposited to GEO and are going to be publicly available after publication. GEO: GSE274613; Other original data reported in this paper will be shared by the lead contact upon request. This study does not report original code. Any additional information required to re-analyze the data reported in this paper is available from the lead contact upon request.
