# Supplementary material for: Molecular insights into the role of desmin intermediate filament network in chromatin landscape, cardiomyocyte differentiation, and maturation
Source: Cell Death Dis. 2025 Oct 16;16(1):723. doi: 10.1038/s41419-025-08056-3 (PMC12533021; doi:10.1038/s41419-025-08056-3)
Supplement: Supplementary file 1 — Supplemental figure legends [file 41419_2025_8056_MOESM1_ESM.docx]

**Supplemental Figure legends**

**Figure S1: Desmin deficiency suppresses the upregulation of Notch1 and Yap pathways and during embryogenesis**

(A) RT-PCR analysis of Notch1 and Yap mRNA levels of adult hearts from 3-month-old mice that were either under basal conditions (Non Swim) or under swimming exercise stress (Swim) conditions in Des^+/+^ and Des^-/-^ backgrounds.

(B) Protein analysis (western blot) of total heart lysates for Notch1 and Yap levels in adult 3-month-old mice under the conditions mentioned in (A) (right panel). Quantitation of protein levels using Quantity One 1-D analysis software (left panel).

(C) Protein analysis (western blot) of cellular fractions of heart lysates for Notch1 (right-top) and Yap (left-top) levels in adult 3-month-old mice under the conditions mentioned in (A). Quantitation of protein levels using Quantity One 1-D analysis software (bottom panel); cyto and nuc: cytoplasmic and nuclear fraction of adult heart lysate, respectively.

**Figure S2: Desmin is expressed in CPCs from early development**

(A) Three-dimensional representation of desmin expression in cardiac progenitor cell (CPC) populations through developmental stages E7.5-E10.5. Color bar indicates the desmin expression levels in the population.

(B) Graphical representation of desmin expression levels in total cardiac cell populations during E7.5-E10.5.

Pdgfra^+^/Gfra2^+^/Kdr^+^*:* CPC markers

**Figure S3: Desmin deficiency affects expression and localization of cardiac proteins**

(A) Relative quantification of ‘high, medium, low’ α-actinin expressing iCMs. Error bars show mean±SEM. **P*<0.2 vs *Des^-/-^*; ***P*<0.1 vs *Des^-/-^*; ****P*<0.05 vs *Des*^−/−^ (Student's unpaired *t*-test).

(B) Protein expression analysis of Connexin 43 by immunoblotting in iCM*^Des+/+^* and iCM*^Des-/-^* 4.5 months after transduction (left panel), quantitation of western analysis (right panel). Connexin 43 shows the most dramatic down regulation in the absence of desmin.

(C) Protein expression analysis of desmoplakin by immunofluorescence in iCM*^Des+/+^* and iCM*^Des-/-^* at 3.5 months after transduction.

(D) Relative quantitation of desmoplakin expressing iCMs by immunofluoresence. Error bars show mean±SEM. **P*<0.01 vs *Des^-/-^*.

(E) i) Protein quantification by densitometry analysis of WB in Figure 4D. GAPDH was used as loading control. Plots represent the mean±S.E.M. of n=3 experiments; **P*<0.5 vs iCMs*^Des^*^+/+^, ***P<*0.05 vs iCMs*^Des^*^+/+^, ****P*≤0.005 vs iCMs*^Des^*^+/+^, (unpaired Student's *t*-test). ii) Densitometry analysis of proteins in Figure 4E. GAPDH was used as loading control. Plots represent the mean±S.E.M. of n=3 experiments; **P*<0.2, ***P<*0.05, ****P*≤0.0001 vs *Des+/+*, (unpaired Student's *t*-test).

**Figure S4: Desmin deficiency leads to the formation of shorter cell-to-cell networks**

(A) Stereoscopic analysis of gross morphology of cell-to-cell networks of a live 3-month iCMs cultures of iCMs . i) cell networks of iCMs*^Des+/+^* ii) cell networks of iCMs*^Des-/-^*. n=3

(B) Immunofluorescence analysis of GFP expression in 4% PFA fixed iCMs*^Des+/+^* and iCMs*^Des-/-^* networks. BF: bright field

(C) Relative quantification of the length of the cell networks in A. Statistics by unpaired Student's t-test. Data are presented as mean± SEM, n=3. *, *P*≤0.1 vs iCMs*^Des+/+^*.

iCM: induced cardiomyocytes

**Figure S5: Desmin deficiency alters nuclear morphology**

Representative electron microscopy images of myocardial tissue ultra-structure from 3-month-old mice. Scale bars: 200nm.

**Figure S6: Desmin deficiency alters gene expression and chromatin modification status**

Correlation of the RNA and ChIP analysis on ACM isolated from *Des^+/+^* and *Des^-/-^* mice under basal and swimming stress conditions. (A) Stacked bar chart indicating percentage of genes with changed expression found by RNA seq (log2 fold change >=|0.6| and adjusted pvalue <0.05), corresponding to peaks found in H3K27Ac ChIP seq upon different backgrounds and stress (swimming) conditions. Number of genes indicated in table chart. (B) GO annotation of the top groups of these altered genes that correspond to common peaks between *Des^+/+^* and *Des^-/-^* background. left panel: Non Swim condition, right panel: Swim condition.

p-adust: adjust p-value= negative log of 10, counts: number of genes in each category

**Figure S7: Desmin influences lamina interacting chromatin**

LaminA/C ChIP was performed in ACM isolated from *Des^+/+^* and *Des^-/-^* mice under basal (A) and stress conditions (B). Top panel: Venn diagrams displaying unique and common peaks on the *Des^+/+^* and *Des^-/-^* backgrounds under basal (A) and stress (B) conditions. Bottom panels: GO annotation analysis showing the most significantly altered groups of genes that correspond to unique peaks in *Des^+/+^* and *Des^-/-^* in condition (A) and (B).

p-adjust: adjust p-value= negative log of 10, counts: number of genes in each category

**Figure S8: Representation of proximal enhancers**

Schematic representation of proximal promoters of the genes: (a) *desmin*, (b) *Notch1*, (c) *connexin 43* (d) α*-actinin* and (e) *desmoplakin*. The region where the enhancers lay and the location of the genes are indicated. Information is obtained from “enhancer atlas 2.0” (http://www.enhanceratlas.org)

**Figure S9**: **Desmin deficiency affects chromatin interaction compartments of cardiomyocytes at a megabase scale.**

Domain insulated regions at (specified genes) locus, obtained by FAN-C analysis, showing triangular Hi-C matrix plots for the conditions mentioned in Fig 8A. Shown: Notch2 (chr3: 97,817,461-97,954,290), VDAC (chr11:52,360,860-52,389,397), Pdgfra and Kdr are located (chr5:75,152,306-75,198,215 and chr5:75,932,827-75,978,458), Long light blue colored boxes cover the location of the gene.
