# Supplementary figures and images for: Molecular insights into the role of desmin intermediate filament network in chromatin landscape, cardiomyocyte differentiation, and maturation
Source: Cell Death Dis. 2025 Oct 16;16(1):723. doi: 10.1038/s41419-025-08056-3 (PMC12533021; doi:10.1038/s41419-025-08056-3)

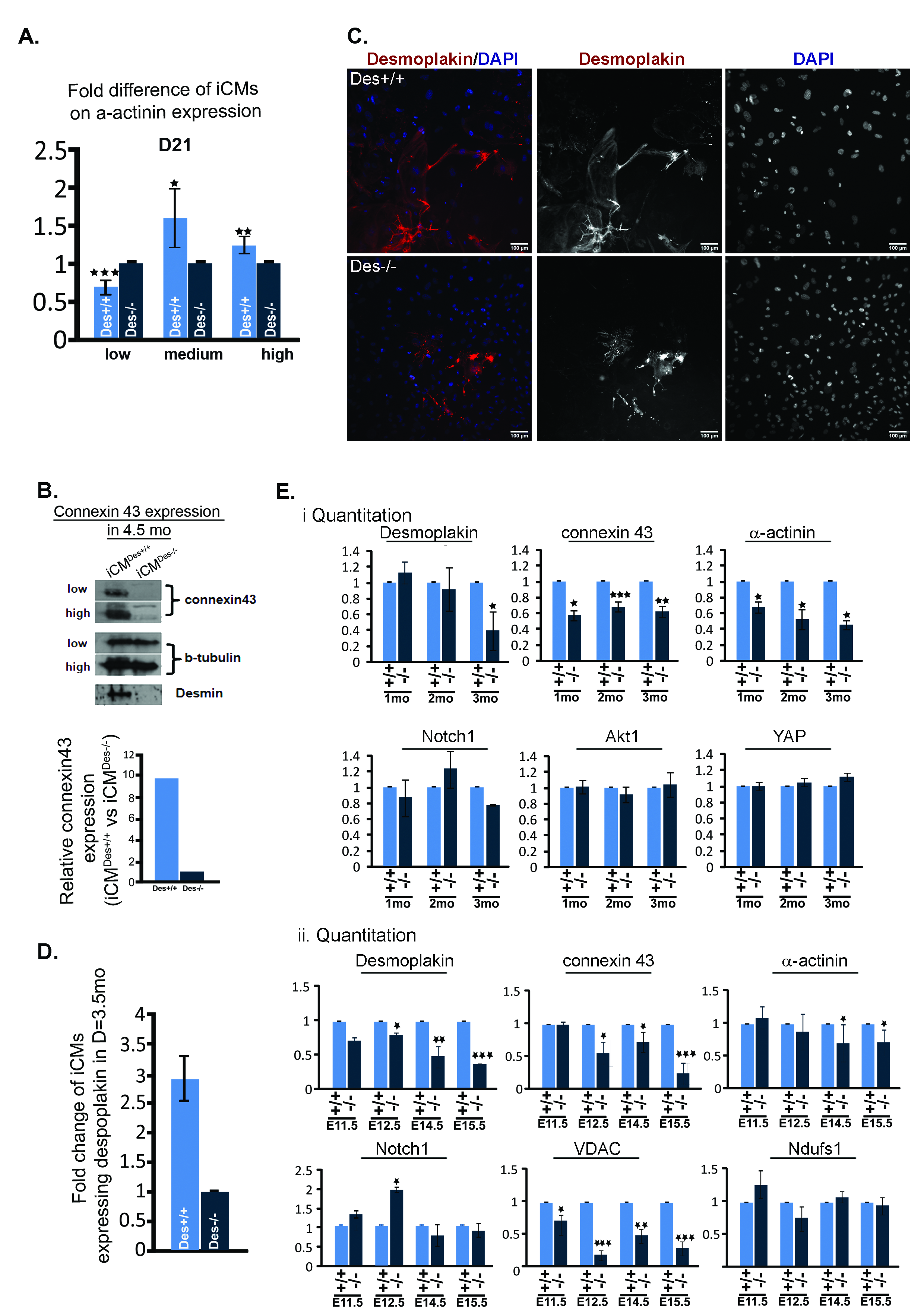

Supplement: Supplementary file 4 — Supplemental Figure 3 [file 41419_2025_8056_MOESM4_ESM.tif]

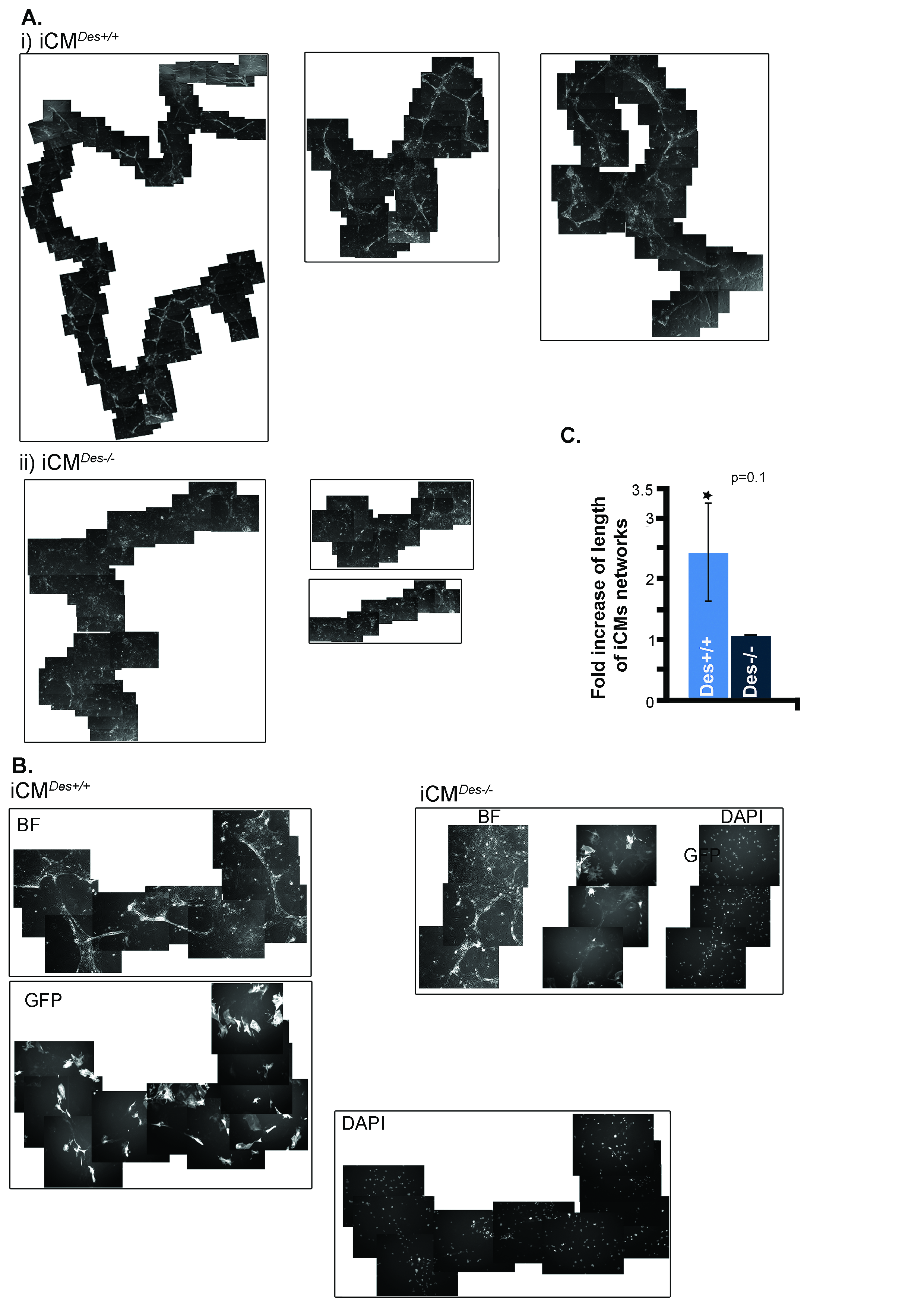

Supplement: Supplementary file 5 — Supplemental Figure 4 [file 41419_2025_8056_MOESM5_ESM.tif]

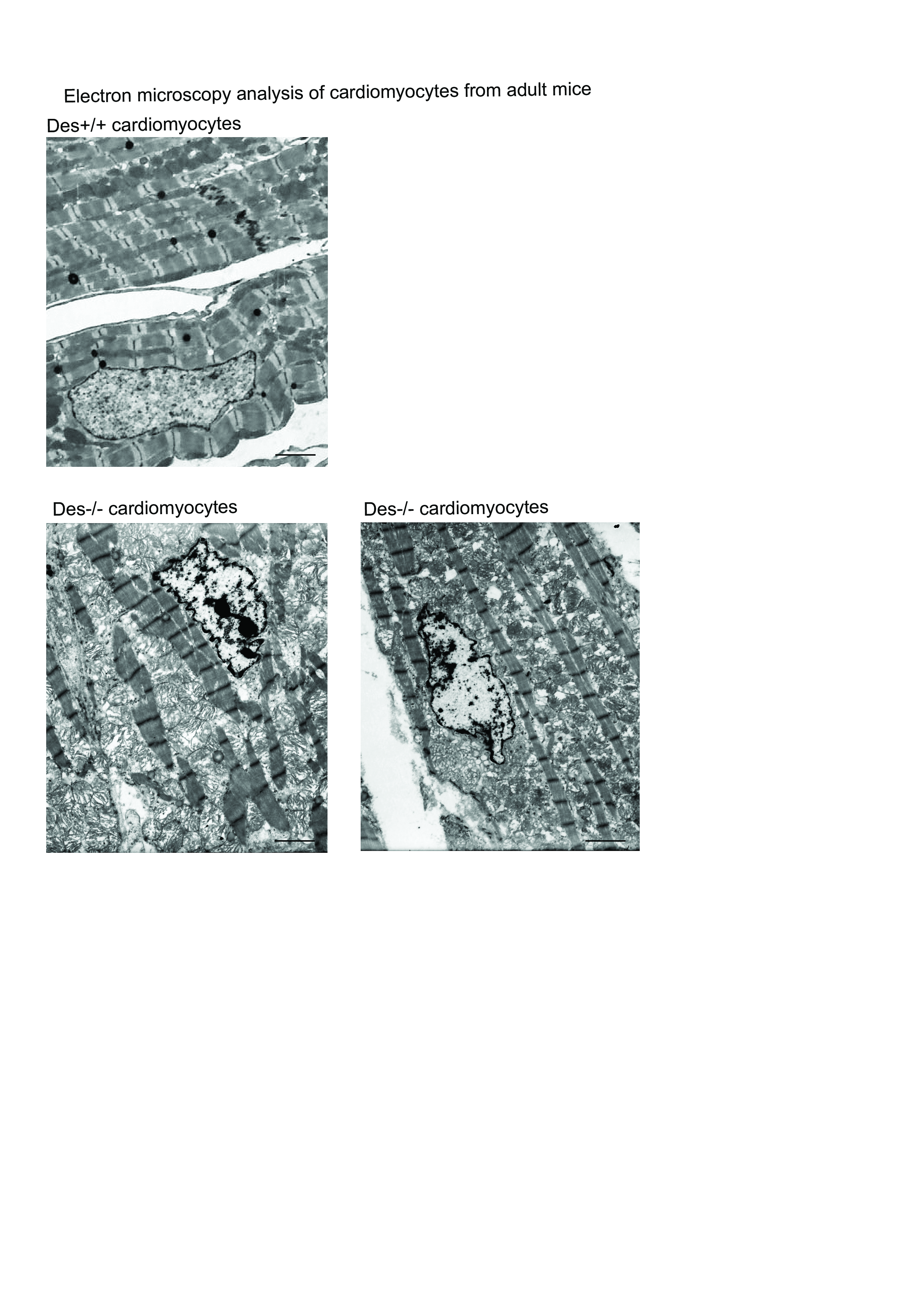

Supplement: Supplementary file 6 — Supplemental Figure 5 [file 41419_2025_8056_MOESM6_ESM.tif]
