# Supplementary material for: Molecular insights into the role of desmin intermediate filament network in chromatin landscape, cardiomyocyte differentiation, and maturation
Source: Cell Death Dis. 2025 Oct 16;16(1):723. doi: 10.1038/s41419-025-08056-3 (PMC12533021; doi:10.1038/s41419-025-08056-3)
Supplement: Supplementary file 11 — Supplemental Table [file 41419_2025_8056_MOESM11_ESM.pdf]

Supplemental Table 1 (reagents)

Reagent	Source	Intentifier
**Antibodies**		
Mouse monoclonal anti- α-actinin (sarcomeric) clone EA-53	Sigma	Product numberA7811
Rabbit polyclonal anti- desmoplakin	Invitrogen	Cat# PA596741
Rabbit polyclonal anti- desmin (H76)	Santa Cruz Biotechnology	Cat# sc-14026
Rabbit polyclonal anti-connexin 43	Sigma	Product number C62191
Rabbit polyclonal anti-LaminA/C	Santa Cruz Biotechnology	Cat# sc-20681
Chicken polyclonal anti-GFP	Abcam	Cat# ab3970
Rat monoclonal anti-Notch1 (5B5)	Cell Signaling	Cat# 3447
Rabbit monoclonal anti-VDAC	Cell Signaling	Cat# 4661
Rabbit polyclonal anti-YAP1	Novusbio	Cat# NB-58358
Rabbit polyclonal anti-YAP1	Cell Signaling	Cat# 14074
Goat polyclonal anti-NDUFS1	ThermoScientific	Cat# PA5-19341
Mouse monoclonal anti-SDHA	Abcam	Cat# ab14715
Rabbit polyclonal anti-Akt	Cell Signaling	Cat# 9272
Rabbit monoclonal anti--β tubulin (9F3)	Cell Signaling	Cat# 2128
Mouse monoclonal anti-GAPDH	Invitrogen	Cat# AM300
Alexa Fluor 647anti-mouse CD309 (VEGFR2, Flk-1)	Biolegend	Cat# 121909
Rat monoclonal PE anti-PDGFR alpha [APA5]	Abcam	Cat# ab93531
Rabbit polyclonal Alexa Fluor 488 anti-desmin [Y66]	Abcam	Cat# ab185033
Goat polyclonal IgG anti-GFRA2	R&D Systems	Cat# AF429
Donkey polyclonal anti-goat UV conjugated IgG Alexa Fluor 405	Abcam	Cat# ab175664
Goat anti-chicken 488	Jackson Immunoresearch	Cat# 103-545-155
Donkey anti-Rabbit Alexa Fluor 594	Life Technologies	Cat# A21207
Donkey anti-Mouse Alexa Fluor 594	Life Technologies	Cat# A21203
Donkey anti-Rabbit Alexa Fluor 488	Life Technologies	Cat# A11034
Rabbit polyclonal anti-LaminA/C (ChIP grade)	Millipore	MABT 1341
Rabbit polyclonal anti-H3K27Ac (ChIP grade)	Abcam	Cat# ab4729

**Experimental models: Cell lines**		
Platinum-E (Plat-E) Retroviral Packaging Cell Line	Cell biolabs, inc.	Cat# RV-101
**Experimental models: DNA plasmids**		

pMXs_Gata4	UCLA: Dr. Srivastava laboratory	Ieda et al[33]
pMXs_Mef2C	UCLA: Dr. Srivastava laboratory	Ieda et al[33]
pMXs_Tbx5	UCLA: Dr. Srivastava laboratory	Ieda et al[33]
pMXs_Desmin	This study	NA
pMXs_DsRed	This study	NA
