# Supplementary material for: Molecular insights into the role of desmin intermediate filament network in chromatin landscape, cardiomyocyte differentiation, and maturation
Source: Cell Death Dis. 2025 Oct 16;16(1):723. doi: 10.1038/s41419-025-08056-3 (PMC12533021; doi:10.1038/s41419-025-08056-3)

Uncut western gels for Figure S1

Uncut western gels for Figure S1 B

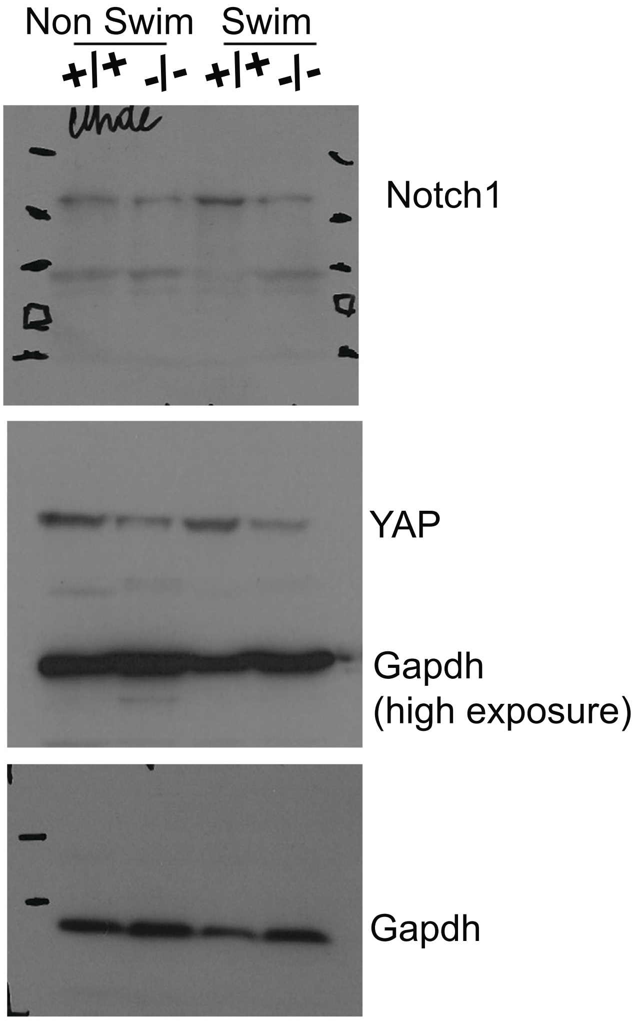

Uncut western gels for Figure S1 C Notch1

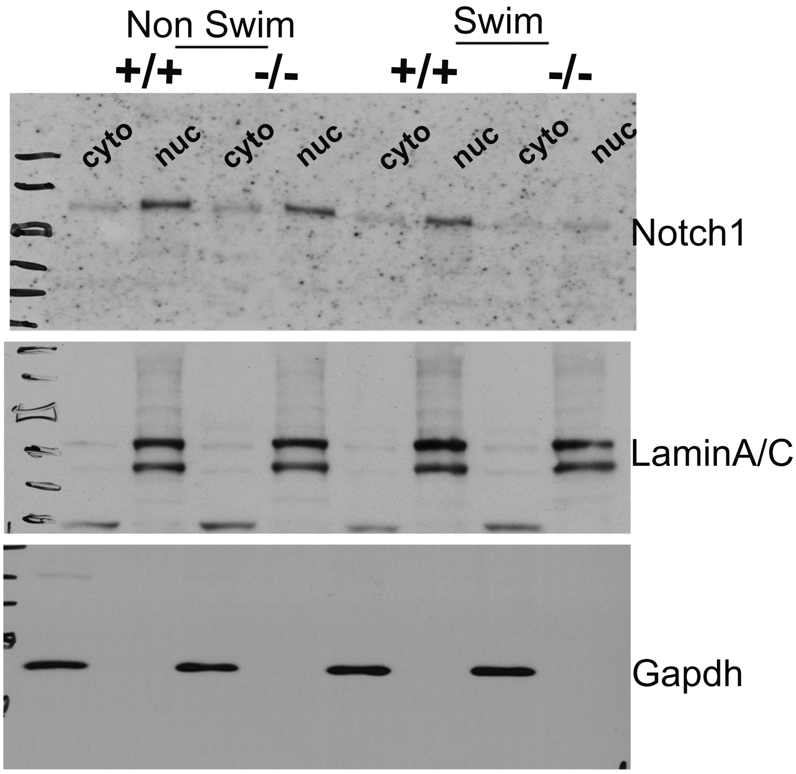

Uncut western gels for Figure S1 C YAP

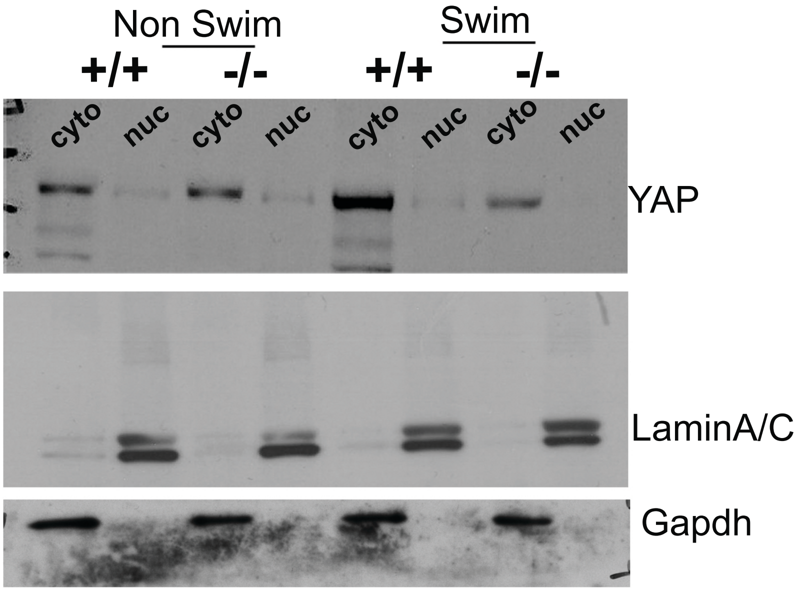

Uncut western gels for Figure 4D

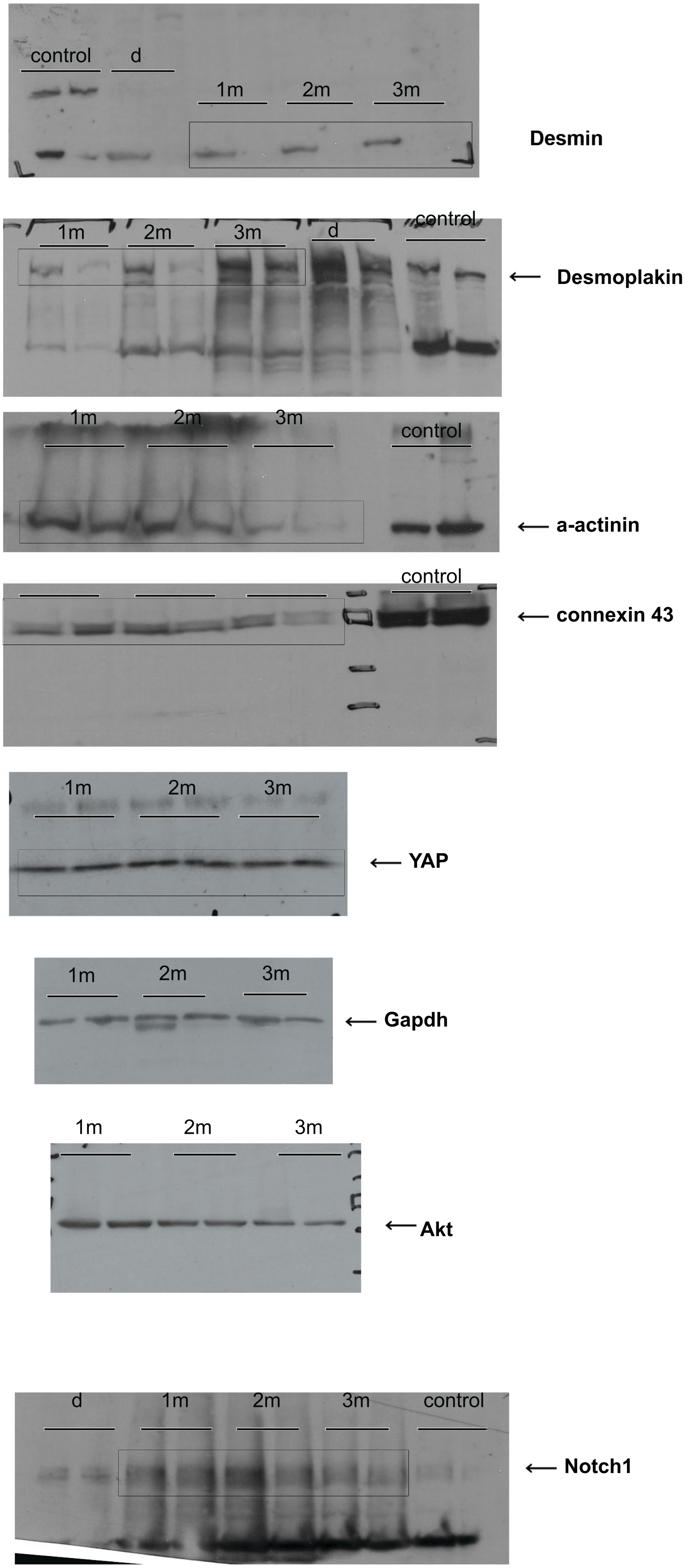

Uncut western gels for Figure 4E,
heart lysates from embryonic day E11.5 and E12.5

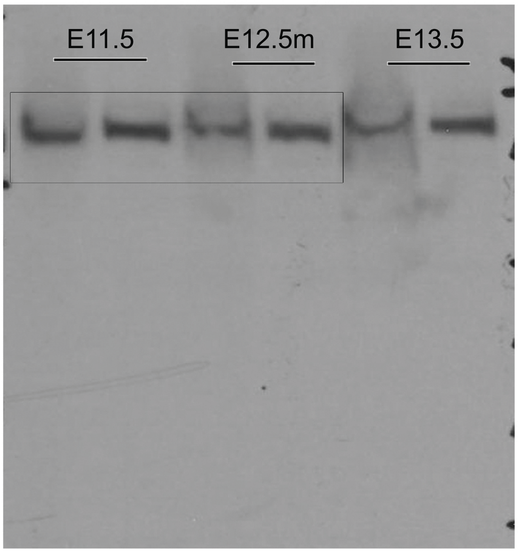

Ndufs1

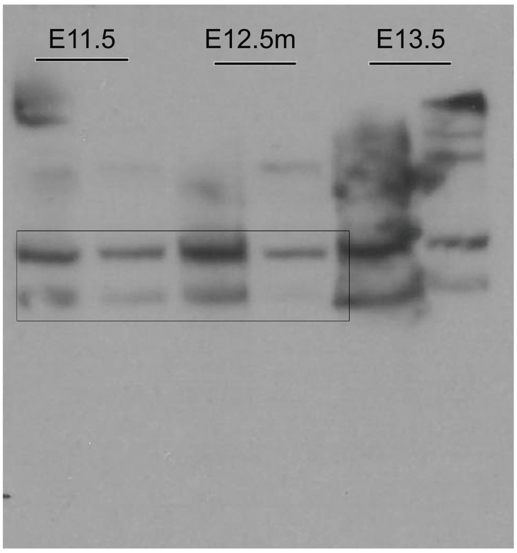

VDAC

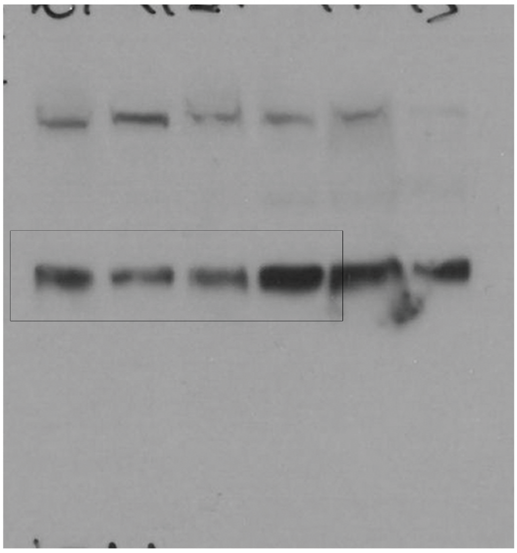

Ndufs1

Gapdh

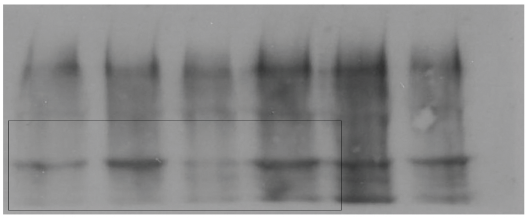

backg

Notch1

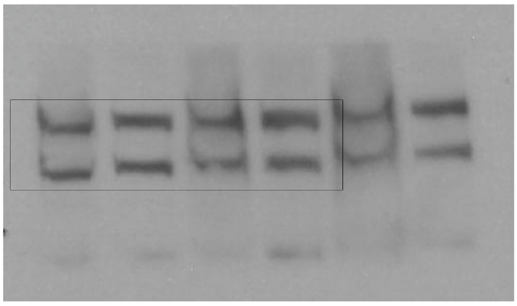

a-actinin

Ndufs1

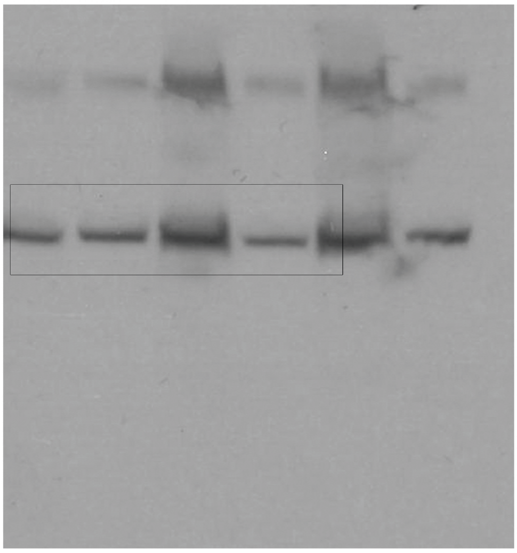

background

connexin 43

Uncut westerns for figure 4E lysates from embyonic hearts E14.5 and E.15.5 (right panel second column)

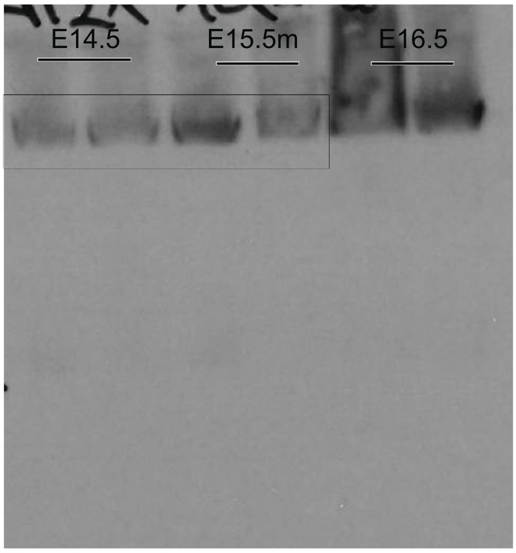

Ndufs1

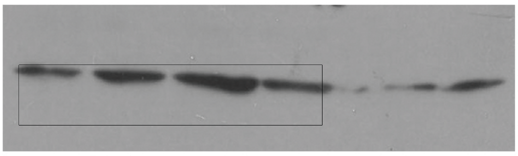

Gapdh

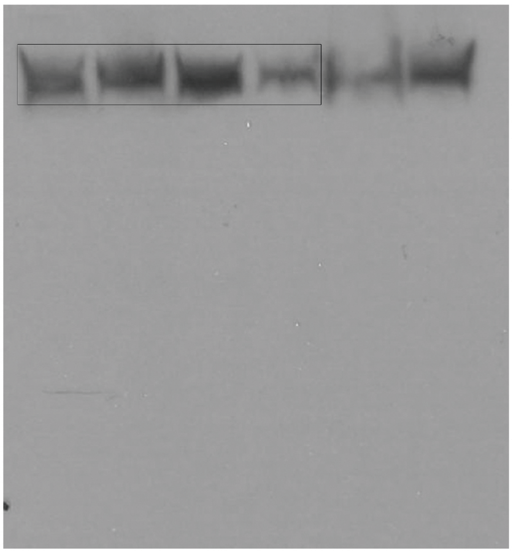

a-actinin

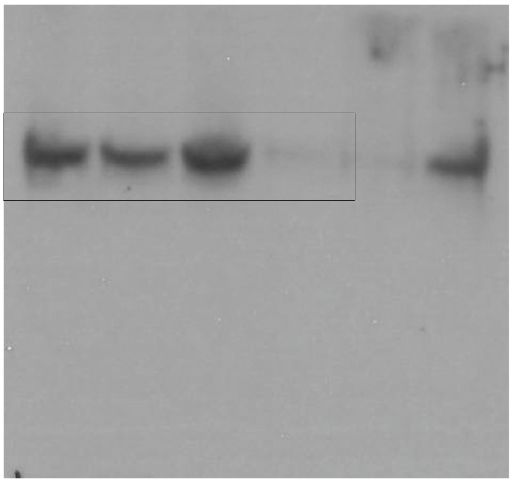

connexin 43

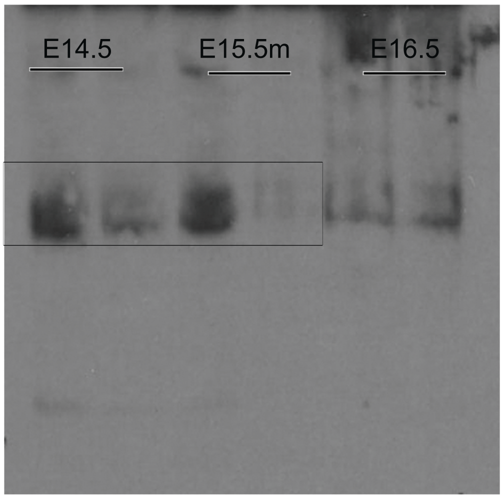

VDAC

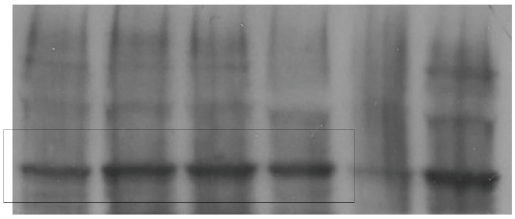

backg

Notch1

Uncut westerns for figure 4E lysates from embyonic hearts E11.5 to E.15.5 rerun

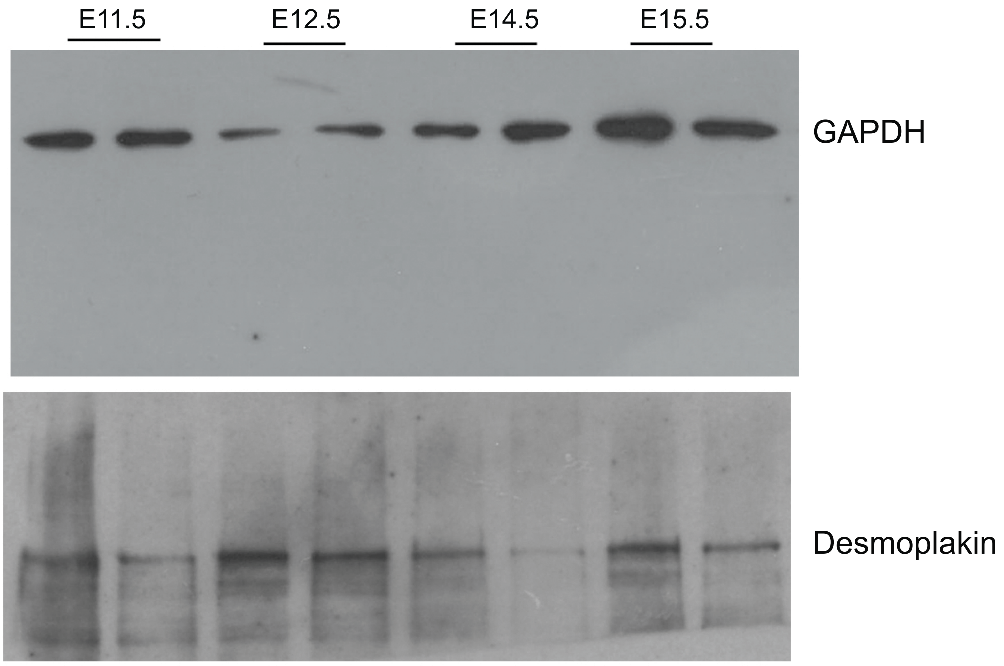

Supplement: Supplementary file 12 — Original western blots of Figures [file 41419_2025_8056_MOESM12_ESM.pdf]
